# B7-H3 suppresses doxorubicin-induced senescence-like growth arrest in colorectal cancer through the AKT/TM4SF1/SIRT1 pathway

**DOI:** 10.1038/s41419-021-03736-2

**Published:** 2021-05-06

**Authors:** Ruoqin Wang, Linqing Sun, Suhua Xia, Hongya Wu, Yanchao Ma, Shenghua Zhan, Guangbo Zhang, Xueguang Zhang, Tongguo Shi, Weichang Chen

**Affiliations:** 1 Jiangsu Institute of Clinical Immunology, The First Affiliated Hospital of Soochow University, 708 Renmin Road, Suzhou, China; 2 Department of Gastroenterology, The First Affiliated Hospital of Soochow University, 188 Shizi Road, Suzhou, China; 3 Jiangsu Key Laboratory of Clinical Immunology, Soochow University, 708 Renmin Road, Suzhou, China; 4 Suzhou Key Laboratory for Tumor Immunology of Digestive Tract, The First Affiliated Hospital of Soochow University, 708 Renmin Road, Suzhou, China; 5 Department of Oncology, The First Affiliated Hospital of Soochow University, 188 Shizi Road, Suzhou, China; 6 Jiangsu Key Laboratory of Gastrointestinal Tumor Immunology, The First Affiliated Hospital of Soochow University, 708 Renmin Road, Suzhou, China

**Keywords:** Cancer therapy, Drug discovery

## Abstract

Emerging evidence suggests that cellular senescence induced by chemotherapy has been recognized as a new weapon for cancer therapy. This study aimed to research novel functions of B7-H3 in cellular senescence induced by a low dose of doxorubicin (DOX) in colorectal cancer (CRC). Here, our results demonstrated that B7-H3 knockdown promoted, while B7-H3 overexpression inhibited, DOX-induced cellular senescence. B7-H3 knockdown dramatically enhanced the growth arrest of CRC cells after low-dose DOX treatment, but B7-H3 overexpression had the opposite effect. By RNA-seq analysis and western blot, we showed that B7-H3 prevented cellular senescence and growth arrest through the AKT/TM4SF1/SIRT1 pathway. Blocking the AKT/TM4SF1/SIRT1 pathway dramatically reversed B7-H3-induced resistance to cellular senescence. More importantly, B7-H3 inhibited DOX-induced cellular senescence of CRC cells in vivo. Therefore, targeting B7-H3 or the B7-H3/AKT/TM4SF1/SIRT1 pathway might be a new strategy for promoting cellular senescence-like growth arrest during drug treatment in CRC.

## Introduction

Colorectal cancer (CRC) is the third most frequently diagnosed malignancy worldwide^1^. Chemotherapy is a conventional treatment option used to fight against CRC^2^ and has made great contributions to the long-term decline in death rates. Doxorubicin (DOX), a kind of anthracycline drug, is known to be effective against a wide variety of cancers, such as carcinomas, sarcomas, and hematological cancers^3^. Unfortunately, the therapeutic efficacy of DOX is proved to be limited due to its toxicity and resistance mechanisms^3,4^. An increasing number of studies have indicated that low-dose DOX can induce cellular senescence^5,6^, which not only reduces side effects but also prevents tumor growth in vitro and in vivo^7,8^. Hence, an intervention strategy dependent on DOX-induced cellular senescence seems to be a potential option in future CRC treatment.

Cellular senescence, first described by Hayflick and Moorhead, is a physiological phenomenon that involves essentially irreversible growth arrest^9^ and is characterized by morphological alteration, chromatin remodeling, cell cycle arrest, increased senescence-associated β-galactosidase (SA-β-gal) activity, and a senescence-associated secretory phenotype (SASP)^10,11^. Cell senescence exists in various precancerous lesions in humans and mice but is reduced in malignant tumors^12^. As a physiological barrier against tumor initiation and progression, cellular senescence is viewed as a natural defense mechanism against tumor progression^13,14^. Therefore, therapy that evokes tumor cellular senescence may be used as a strategy for tumor treatment. A growing body of work has indicated that multiple abnormally expressed genes in cancer cells may participate in the regulation of tumor cellular senescence induced by drugs^8^. For instance, the chromatin remodeling enzyme ATRX is a critical regulator of therapy-induced senescence^15^. The knockdown of TRIB2, a member of the tribble family, promoted DOX-induced senescence of SW48 and LoVo CRC cells in a p21-dependent manner^16^. Zhu and coworkers showed that nuclear receptor HNF4α overexpression enhanced cellular senescence caused by DOX in prostate cancer cells^17^. Nevertheless, the underlying molecular mechanism of the regulation of DOX-induced senescence has not yet been well elucidated.

B7-H3 (B7 homolog 3), also known as CD276, serves as a member of the B7-CD28 superfamily^18^ and is overexpressed in a variety of malignancies, such as CRC, pancreatic cancer, prostate cancer, and ovarian cancer^19–22^. Due to conflicting costimulatory and coinhibitory functions, the immunologic function of B7-H3 remains controversial^23^. Aside from its immunologic function, B7-H3 has been reported to be involved in tumorigenesis-associated nonimmunological functions such as proliferation, metastasis, metabolism, and angiogenesis^24–27^. Moreover, B7-H3 exerts a great impact on the regulation of chemoresistance in several malignancies, including CRC^28,29^. Although senescence-associated exosomes of human prostate cancer cells are enriched in the B7-H3 protein^30^, few studies have explored the detailed roles and molecular mechanisms of B7-H3 in modulating cellular senescence in cancers such as CRC. Hence, the aim of the current study was to explore whether B7-H3 regulated cellular senescence and growth arrest in CRC cells treated with a low dose of DOX in an attempt to optimize chemotherapy-induced senescence therapies associated with B7-H3.

## Materials and methods

### CRC clinical samples and immunohistochemistry (IHC) assay

A total of 54 pairs of CRC primary tumor tissues and adjacent normal tissues were obtained from CRC patients who received surgical resection at the First Affiliated Hospital of Soochow University (Suzhou, China). This study was approved by the Institutional Review Board of Soochow University, and informed consent was obtained from the patients. Detailed clinicopathological information is provided in Supplementary Table 1. An IHC assay and the scoring criteria were conducted as previously described^26^. Briefly, after deparaffinization and rehydration, tissues were treated with antigen retrieval using 10 mM sodium citrate buffer (pH 6.0), followed by incubation with B7-H3 antibody (R&D Systems, MN, USA, #AF1027, 1:100) and TM4SF1 antibody (Abcam, Cambridge, UK, #ab113504, 1:500) overnight at 4 °C. We used the semiquantitative immunoreactive score (IRS) system to analyze the B7-H3 and TM4SF1 immunostaining. The intensity of immunostaining was scored using the following criteria: 0, negative; 1, weak; 2, moderate; 3, strong. Besides, the percentage of immunoreactive cells was scored using the following criteria:1, (0–25%); 2, (26–50%); 3, (51–75%); and 4, (76–100%). Final scores were calculated by multiplying the scores of two parts in the same section; the scores ranged from 0 to 12. Two pathologists (Dr. Xia and Dr. Zhan) reviewed blindly and scored IHC staining independently for each sample.

### Cell culture, transfection, and infection

The cell lines HCT116 and RKO were obtained from the American Type Culture Collection (ATCC, Manassas, VA, USA). These cells were cultured in DMEM (Biological Industries, Beit Haemek, Israel) plus 10% fetal bovine serum (FBS, Biological Industries) and 1% penicillin-streptomycin (Beyotime, Shanghai, China, #C0222) at 5% CO_2_ and 37 °C.

Cells were transfected with TM4SF1 siRNA, SIRT1 siRNA or control siRNA (RiboBio, Guangzhou, China) using Lipofectamine 2000 (Invitrogen, Carlsbad, CA, USA) according to the manufacturer’s protocol. The TM4SF1 plasmid was obtained from GeneCopoeia (Guangzhou, China, #EX-Z2186-Lv105). TM4SF1-overexpressing HCT116 or RKO cells were generated according to the manufacturer’s protocol. Stable B7-H3-overexpressing HCT116 or RKO cells (B7-H3 HCT116 or B7-H3 RKO) and knockdown HCT116 or RKO cells (shB7-H3 HCT116 or shB7-H3 RKO) were described earlier^27^.

### Induction of cell senescence

For induction of cell senescence by DOX (Aladdin, Shanghai, China, #25316-40-9), HCT116 and RKO cells were treated with a variety of concentrations of DOX (HCT116: 100 nM, RKO: 50 nM) and cultured in DMEM with 5% FBS for 96 h. The established cellular senescence models were used for further analysis.

### Western blot analysis

Cells were lysed in RIPA lysis buffer (Beyotime, #P0013D) at 4 °C. Protein concentration was examined using the BCA method (Beyotime, #P0010). Total protein (30 μg) was separated via 10% SDS-PAGE (Beyotime, #P0012AC) and transferred onto 0.45-µm PVDF membranes (GE Healthcare Life Science, Germany). After blocking with 5% BSA (Fcmacs, Nanjing, China, #FMS-WB021) for 1 h, the membranes were incubated at 4 °C overnight with one of the antibodies listed in Supplementary Table 2. The next day, membranes were washed and incubated with the corresponding HRP-conjugated secondary antibodies (Beyotime) for 1 h at room temperature. Finally, immunoreactive bands were detected with ECL reagents (NCM Biotech, Suzhou, China, #10100) using a ChemiDocTM MP Imaging System (Bio-Rad).

### RNA extraction and real-time quantitative PCR (RT-qPCR)

Total RNA was isolated from CRC cells using TRIzol (TransGen Biotech, Beijing, China, #ER501-01) in accordance with the manufacturer’s instructions. Then, cDNA was synthesized using PrimeScript RT Master Mix (Takara, Shiga, Japan, #RR036A), and RT-qPCR reactions were performed using SYBR Green Master Mix (Vazyme, Nanjing, China, #Q121–02-AA) according to the manufacturer’s instructions. The reaction conditions were described as previously^27^. β-actin was used as a constitutive control. PCRs for each sample were conducted in triplicate. The primers were designed based on GenBank sequences and are listed in Supplementary Table 3.

### SA-β-gal staining

Cells were subjected to SA-β-gal staining using the Senescent β-galactosidase Staining Kit (Beyotime, #C0602) according to the manufacturer’s protocol. Briefly, cells were washed with PBS and fixed with the fixative solution for 15 min. Then, the cells were incubated with the staining solution overnight at 37 °C without CO_2_. Finally, green-stained positive cells were photographed and counted from three different locations per well by an inverted microscope.

### SAHF detection

Senescence-associated heterochromatin foci (SAHF), visualized as DAPI-dense foci, is a feature of some senescent cells^10^. To identify the SAHFs, cells were fixed with 4% paraformaldehyde for 15 min and permeabilized with 1% Triton X-100 for 10 min. Then, the cells were stained with DAPI (Invitrogen, USA, #S36939) for 5 min. DAPI-stained nuclei with blue fluorescence were finally photographed and counted from three different locations per well under a confocal laser scanning microscope (Olympus FLUOVIEW FV1000).

### CCK-8 assay

Cell viability was determined by using the Cell Counting Kit-8 (Dojindo, Kumamoto, Japan, #CK04) assay. Briefly, CRC cells were seeded in 96-well plates (5 × 10^3^ cells/well). After the cells were treated with DOX for 24, 48, 72 or 96 h, 10 µl CCK-8 solution was added into each well for 4 h at 37 °C. The absorbance in each well was measured at a wavelength of 450 nm.

### Colony formation assay

For the colony formation assay, cells were seeded into 6-well plates (2 × 10^3^ cells/well) and incubated for 14 days. The colonies were fixed in 4% paraformaldehyde for 15 min, washed with PBS, and stained with crystal violet (Beyotime, #C0121) for 15 min. Finally, the number of colonies was photographed and counted from three different wells.

### Cell cycle analysis

Cells were collected, washed with cold PBS twice and fixed with 70% ethanol containing 0.5% FBS overnight at −20 °C. Cells were then treated with cell cycle assays (Fcmacs, Nanjing, China) according to the manufacturer’s protocol. Cell cycle distribution was measured by the Becton-Dickinson FACScan System (Franklin Lakes, NJ, USA). A total of 10,000 events were collected per sample.

### RNA-seq assay

For RNA-Seq analysis, total RNA was extracted from senescent shB7-H3 RKO cells or control cells as described above. The quantity and quality of the total RNA were determined by measuring the OD 260/280 ratio, and samples with ratios between 1.8 and 2.1 were confirmed as having good integrity. The RNA-seq assay was carried out by GENEWIZ Biotech Co. (Suzhou, China).

### Xenograft tumor

The animal assay was approved by the Institutional Animal Care and Use Committee of Soochow University (Suzhou, China). Six to eight-week-old female BALB/C nude mice were purchased from the Shanghai Laboratory Animal Center (Shanghai, China). All mice were randomly assigned to different groups. To create the xenograft tumor model, 5 × 10^6^ B7-H3 HCT116 cells, EV HCT116 cells, shB7-H3 HCT116 cells or sh-NC HCT116 cells were injected subcutaneously into the right flank of the mice, respectively. To investigate the effect of B7-H3 on DOX-induced cellular senescence in vivo, mice with xenograft tumors were intraperitoneally injected with DOX at a low dose of 4 mg/kg every other day. The volume of xenograft tumors was measured every 3 days by using digital calipers. After 2 weeks, all mice were sacrificed, and tumor samples were weighed. In addition, the xenograft tumor tissues were studied by SA-β-gal staining and IHC assay. All results of animal experiments were obtained blindly.

### Statistical analysis

All calculations were analyzed using GraphPad Prism 5.0 (La Jolla, CA, USA). The significance of differences between two groups was determined by Student’s *t*-test. All data are expressed as at least three biological replicates. A value of *P* < 0.05 was considered statistically significant.

## Results

### B7-H3 suppresses cellular senescence and senescence-like cell growth arrest in CRC cells

**Fig. 1:**
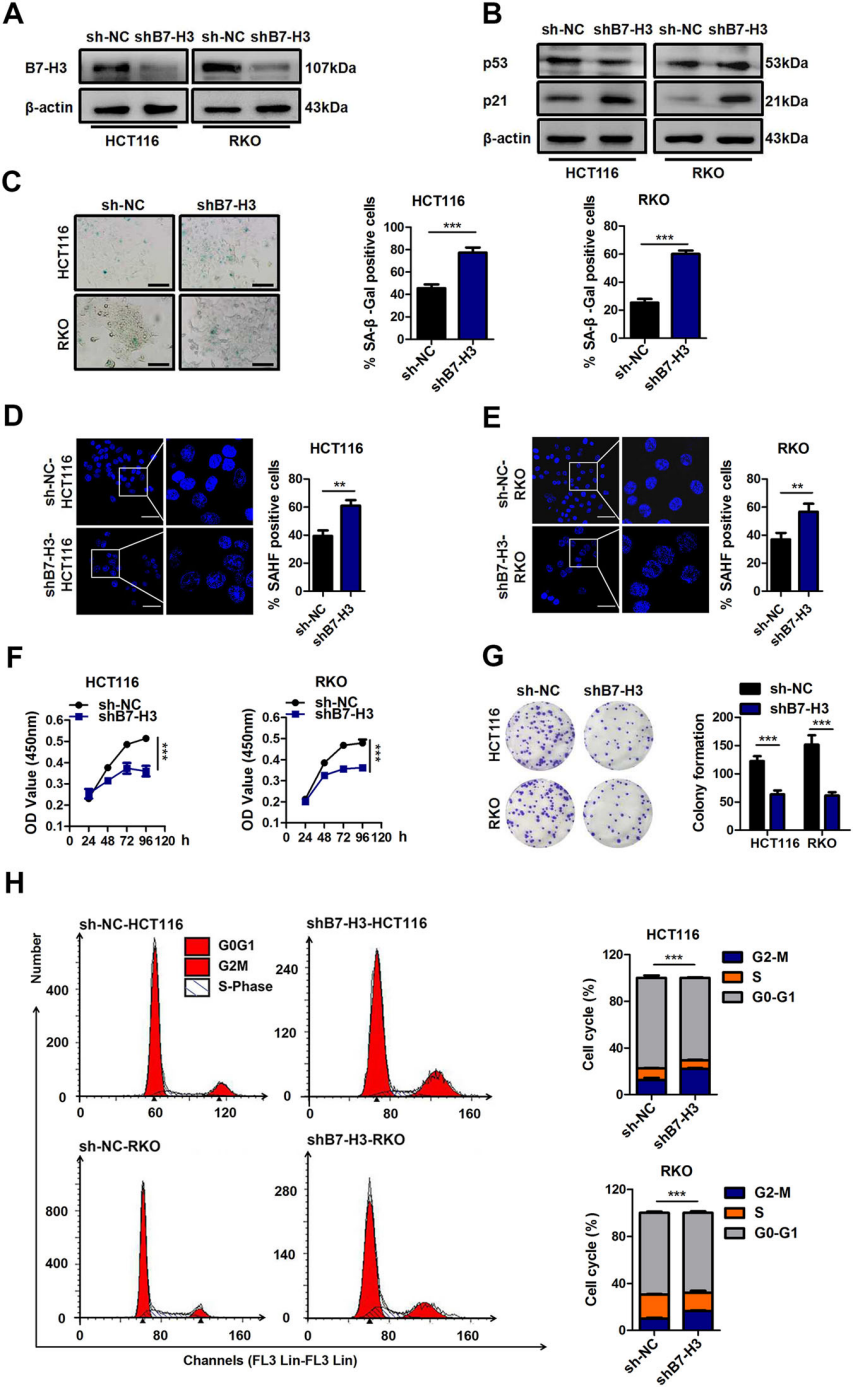
Knockdown of B7-H3 promotes DOX-induced senescence. **A** Western blot analysis of B7-H3 in CRC stable cell lines with B7-H3 inhibition (shB7-H3) or their control cell lines (sh-NC). β-Actin served as a loading control. **B** Western blot analysis of p21 and p53 in sh-NC cells or shB7-H3 CRC cells treated with DOX. β-Actin served as a loading control. **C** SA-β-Gal activity of sh-NC cells or shB7-H3 CRC cells treated with DOX was examined. Scale bar, 100 μm. One representative image from three reproducible experiments is shown. The percentages of SA-β-gal-positive cells are shown in the bar graph. **D**, **E** SAHF activity of sh-NC cells or shB7-H3 HCT116 cells and RKO cells with DOX treatment was examined. Scale bar, 50 μm. One representative image from three reproducible experiments is shown. The percentages of SAHF-positive cells are shown in the bar graph. **f** Cell viability in sh-NC cells or shB7-H3 CRC cells treated with DOX after 24, 36, 48, and 96 h was examined by CCK-8 assays. **G** The colony formation of sh-NC cells or shB7-H3 CRC cells treated with DOX was examined. One representative image from three reproducible experiments is shown. The number of colonies is shown in the bar graph. **H** Cell cycle analysis by PI staining in sh-NC cells or shB7-H3 CRC cells with DOX treatment was examined through flow cytometry. The data represent the means ± SEM. *P < 0.05; ***P < 0.001.

To identify the functional role of B7-H3 in low-dose DOX-induced senescence in CRC cells, we used stable B7-H3 knockdown (shB7-H3) HCT116 or RKO cells that we described previously^27^. B7-H3 knockdown stably inhibited B7-H3 mRNA and protein expression in HCT116 or RKO cells (Supplementary Fig. S1A and Fig. 1A). We treated shB7-H3 HCT116 or RKO cells with a low dose of DOX (100 nM for HCT116 cell, and 50 nM for RKO cell) for 96 h and found that the protein expression of p21 was markedly increased under B7-H3 knockdown conditions (Fig. 1B). B7-H3 knockdown had no effect on the protein expression of p53 in CRC cells treated with low-dose DOX (Fig. 1B). The percentage of SA-β-gal- and SAHF-positive cells was obviously increased in shB7-H3 CRC cells treated with low-dose DOX (Fig. 1C–E). Moreover, we observed that B7-H3 knockdown dramatically enhanced the growth arrest of CRC cells treated with low-dose DOX, as detected by both CCK-8 and colony formation assays (Fig. 1F, G). Additionally, the flow cytometry-based cell cycle assay results showed that B7-H3 knockdown further aggravated G2/M phase arrest in HCT116 and RKO cells treated with low-dose DOX (Fig. 1H).

**Fig. 2:**
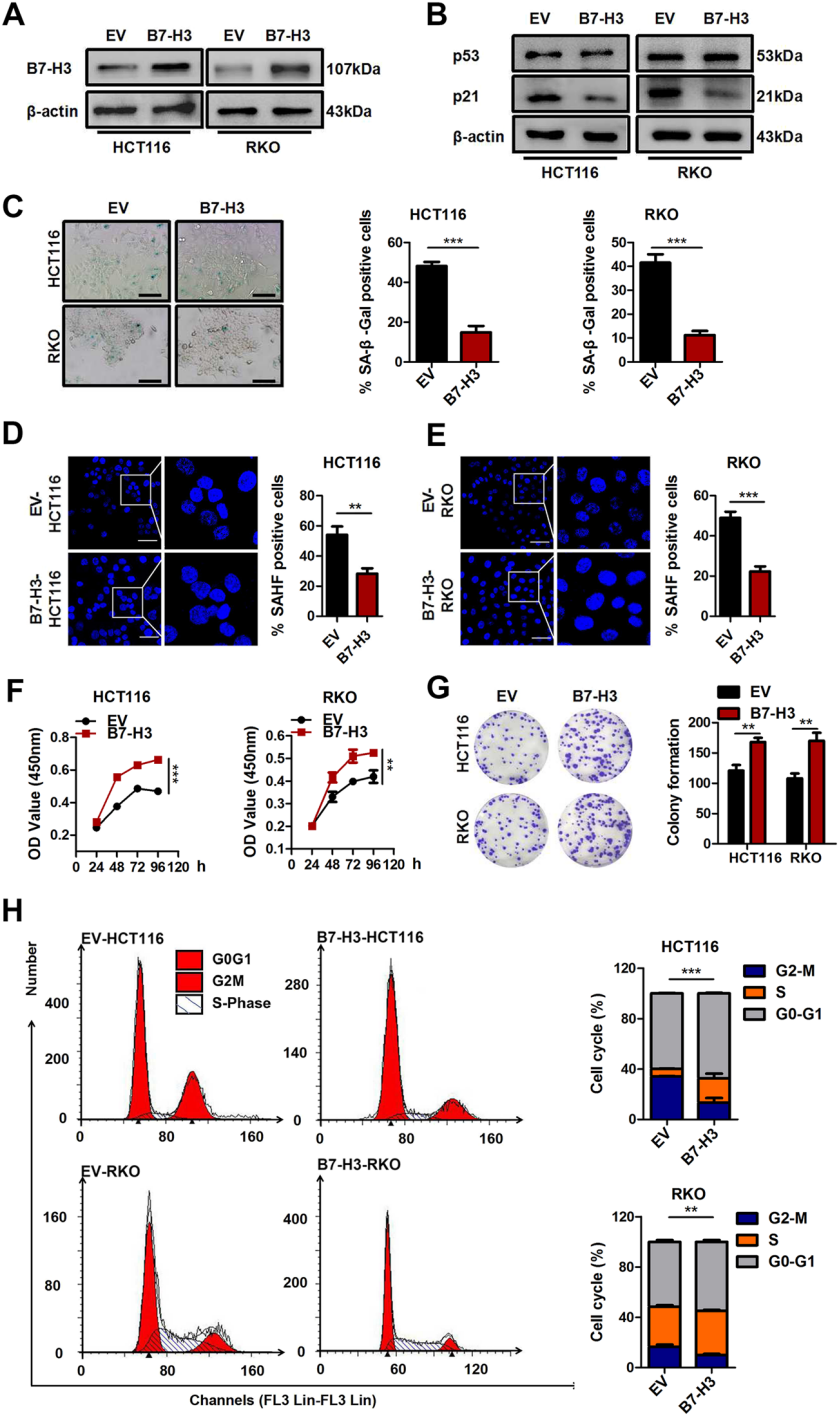
Overexpression of B7-H3 attenuates the DOX-induced senescence. **A** Western blot analysis of B7-H3 in CRC stable cell lines with B7-H3 overexpression (B7-H3) or their control cell lines (EV). β-Actin served as a loading control. **B** Western blot analysis of p21 and p53 in EV cells or B7-H3 CRC cells treated with DOX. β-Actin served as a loading control. **C** SA-β-Gal activity of EV cells or B7-H3 CRC cells treated with DOX was examined. Scale bar, 100 μm. One representative image from three reproducible experiments is shown. The percentages of SA-β-gal-positive cells are shown in the bar graph. **D**, **E** SAHF activity of EV cells or B7-H3 HCT116 cells and RKO cells with DOX treatment was examined. Scale bar, 50 μm. One representative image from three reproducible experiments is shown. The percentages of SAHF-positive cells are shown in the bar graph. **F** Cell viability in EV cells or B7-H3 CRC cells treated with DOX after 24, 36, 48, and 96 h was examined by CCK-8 assays. **G** The colony formation of EV cells or B7-H3 CRC cells treated with DOX was examined. One representative image from three reproducible experiments is shown. The number of colonies is shown in the bar graph. **H** Cell cycle analysis by PI staining in EV cells or B7-H3 CRC cells with DOX treatment was examined through flow cytometry. The data represent the means ± SEM. *P < 0.05; ***P < 0.001.

In complementary loss-of-function studies, HCT116 or RKO cells stably overexpressing B7-H3 were used (Supplementary Fig. S1B and Fig. 2A). B7-H3 overexpression significantly decreased the protein expression of p21 in HCT116 and RKO cells treated with low-dose DOX but had no effect on p53 protein expression (Fig. 2B). Moreover, B7-H3 overexpression obviously reduced the percentage of SA-β-gal- and SAHF-positive CRC cells treated after low-dose DOX treatment (Fig. 2C–E). Furthermore, the effects of B7-H3 overexpression on senescence-like growth arrest and cell cycle arrest were evaluated, and the results revealed that after the elevation of B7-H3, low-dose DOX-induced CRC cell growth arrest and G2/M cell cycle arrest were alleviated (Fig. 2F–H). Based on the obtained results, we conclude that B7-H3 is involved in the resistance to low-dose DOX-induced cell senescence in CRC.

### B7-H3 promotes the expression of TM4SF1 in senescent CRC cells

**Fig. 3:**
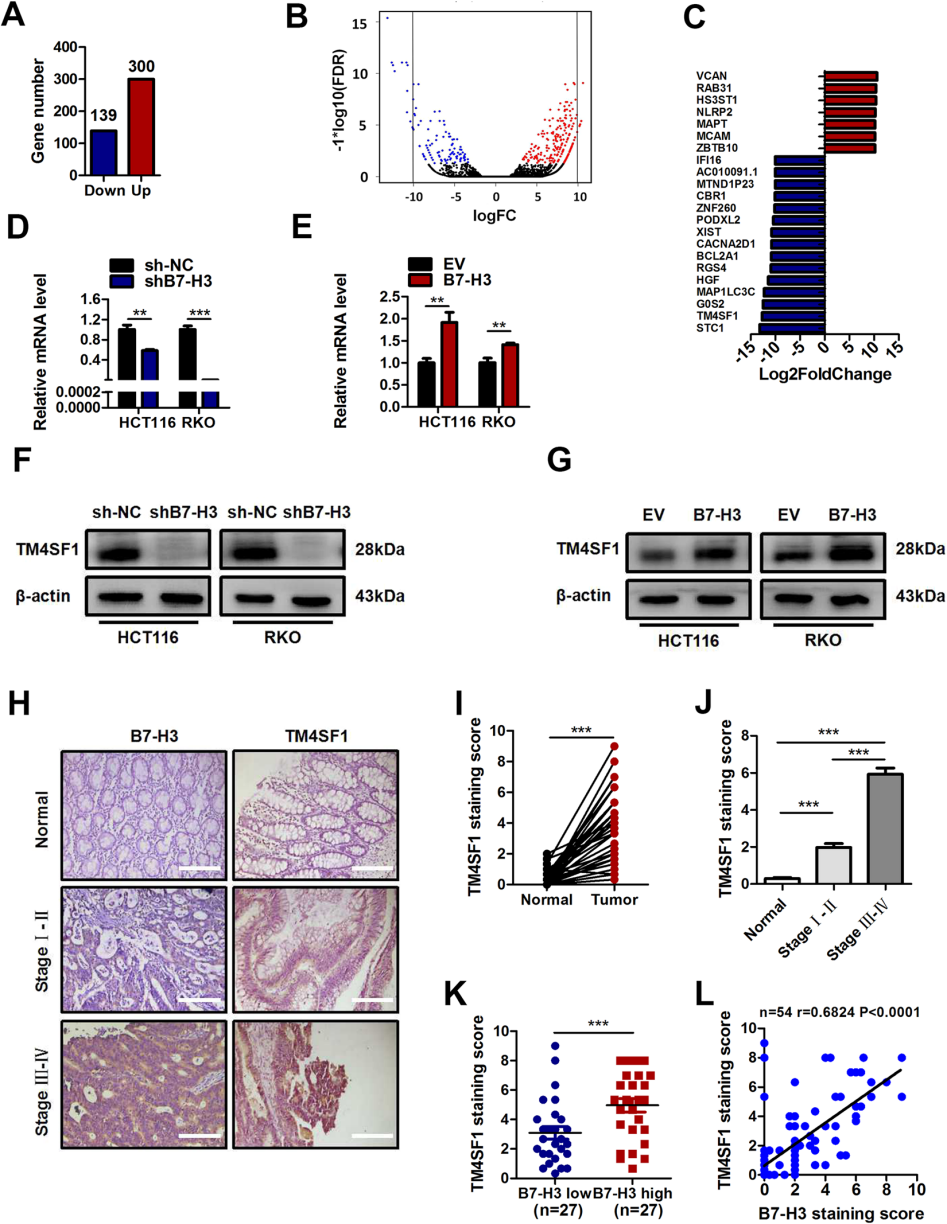
B7-H3 promotes the expression of TM4SF1 in DOX-induced senescence. **A** RNA sequencing (RNA-seq) analysis showed the number of differentially expressed genes (DEGs) (139 downregulated and 300 upregulated) in sh-NC cells or shB7-H3 RKO cells with DOX treatment. **B** Image of RNA-Seq volcano plot showing logarithm of fold change in DEGs. **C** DEGs with logarithm of fold change (|logFC|) > 10 and *P*-value < 0.05 were identified. **D** The mRNA expression of TM4SF1 in sh-NC cells or shB7-H3 CRC cells treated with DOX. **E** The mRNA expression of TM4SF1 in EV cells or B7-H3 CRC cells treated with DOX. **F** Western blot analysis of TM4SF1 and p21 in sh-NC cells or shB7-H3 CRC cells treated with DOX. β-Actin served as a loading control. **G** Western blot analysis of TM4SF1 and p21 in EV cells or B7-H3 CRC cells treated with DOX. β-Actin served as a loading control. **H** Representative images of IHC for B7-H3 and TM4SF1 in CRC tissues and matched normal tissues from 54 clinical CRC patients. Scale bar, 100 μm. **I** TM4SF1 protein expression based on the staining index of CRC specimens and matched normal tissues. **J** TM4SF1 protein expression based on staining index in CRC specimens at different clinical stages. **K** TM4SF1 protein expression is shown for patients stratified into B7-H3 low (<median value) and B7-H3 high (>median value) groups. **L** Correlation analysis of the staining scores (protein expression levels) of B7-H3 and TM4SF1 in human CRC specimens (*n* = 54). The correlation coefficient (*r*) is shown. The data represent the means ± SEM. **P < 0.01; ***P < 0.001.

To determine the reasons why B7-H3 inhibited cellular senescence and senescence-like growth arrest in CRC, we performed RNA-seq analysis to identify the differentially expressed genes (DEGs) in shB7-H3 RKO cells treated with low-dose DOX. First, DEG analysis yielded a list of 439 genes that were significantly dysregulated (139 downregulated and 300 upregulated, *P* < 0.05, Fig. 3A). Among these, 22 DEGs with logarithm of fold change (|logFC|) > 10 were identified (Fig. 3B, C and Supplementary Table 4). Then, we noticed transmembrane-4 L-six family member-1 (TM4SF1) in the top differentially expressed genes. TM4SF1, a signal transducer, has been reported to regulate cell proliferation, migration, invasion, and chemoresistance in multiple cancers^31–33^. Therefore, we hypothesized that B7-H3 might affect the cellular senescence of CRC cells by modulating TM4SF1. The results of RT-qPCR and western blot assays showed that the mRNA and protein expression of TM4SF1 were significantly downregulated in shB7-H3 CRC cells but upregulated in B7-H3-overexpressing cells (Fig. 3D–G). To conclude, TM4SF1 does factor into B7-H3-associated resistance to low-dose DOX-induced senescence.

### TM4SF1 is highly expressed and positively correlates with B7-H3 expression in CRC clinical tissues

Next, we investigated the correlation between the levels of B7-H3 and TM4SF1 in CRC clinical samples by IHC staining. Clearly, TM4SF1, the same as B7-H3, was strongly upregulated in CRC tissue samples compared to that in normal tissues (Fig. 3H, I and Supplementary Fig. S2A). Additionally, the expression of TM4SF1 was increased in advanced (III and IV stages) CRC patients compared to early stage (I and II) patients (Fig. 3J), sharing the same tendency with the expression of B7-H3 (Supplementary Fig. S2B). Moreover, we selected 27 cases (50%) with low expression of B7-H3 (<median value) and 27 cases (50%) with high expression (>median value). The high B7-H3 expression group showed higher TM4SF1 expression than the low group (Fig. 3K). More importantly, the expression level of TM4SF1 showed an obvious positive association with B7-H3 levels in CRC tissue samples (Fig. 3L). In addition, samples of CRC with lymph node metastasis showed higher B7-H3 and TM4SF1 expression than those without lymph node metastasis (Supplementary Fig. S2C, D).

### B7-H3 regulates cellular senescence via TM4SF1

**Fig. 4:**
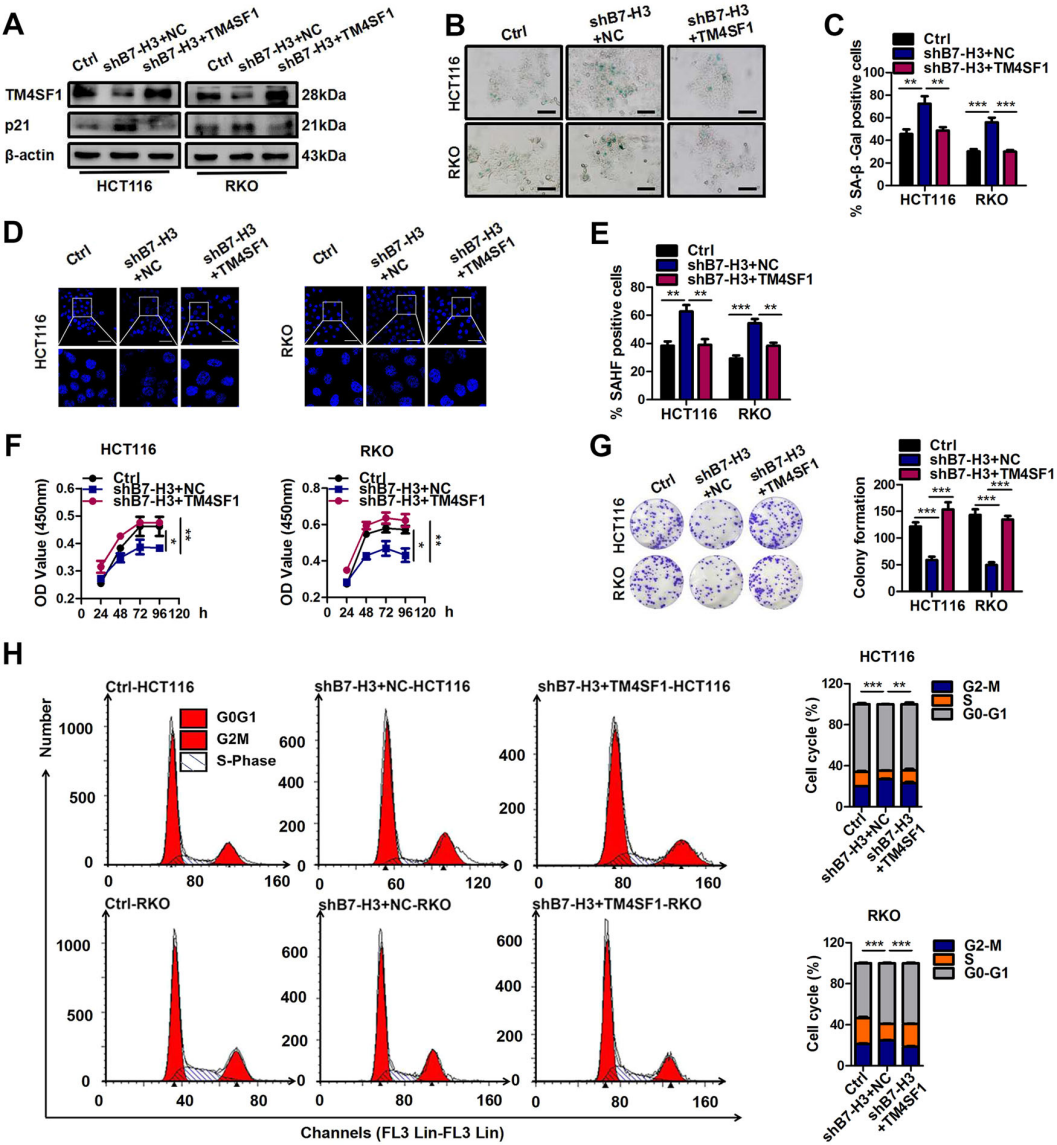
TM4SF1 is involved in B7-H3-associated resistance to DOX-induced senescence. **A** Western blot analysis of TM4SF1, and p21 in control cells or shB7-H3 CRC cells after treatment with TM4SF1 overexpression vectors and DOX. β-Actin served as a loading control. **B**, **C** SA-β-Gal activity of control cells or shB7-H3 CRC cells after treatment with TM4SF1 overexpression vectors and DOX was examined. Scale bar, 100 μm. One representative image from three reproducible experiments is shown. The percentages of SA-β-gal-positive cells are shown in the bar graph. **D**, **E** SAHF activity of control cells or shB7-H3 CRC cells after treatment with TM4SF1 overexpression vectors and DOX was examined. Scale bar, 50 μm. One representative image from three replicate experiments is shown. The percentages of SAHF-positive cells are shown in the bar graph. **F** The cell viability of control cells or shB7-H3 CRC cells with TM4SF1 overexpression vectors and DOX treatment after 24, 36, 48, and 96 h was examined by CCK-8 assays. **G** The colony formation of control cells or shB7-H3 CRC cells after treatment with TM4SF1 overexpression vectors and DOX was examined. One representative image from three reproducible experiments is shown. The number of colonies is shown in the bar graph. **H** Cell cycle analysis by PI staining in control cells or shB7-H3 CRC cells after treatment with TM4SF1 overexpression vectors and DOX was examined through flow cytometry. The data represent the means ± SEM. *P < 0.05; **P < 0.01; ***P < 0.001.

**Fig. 5:**
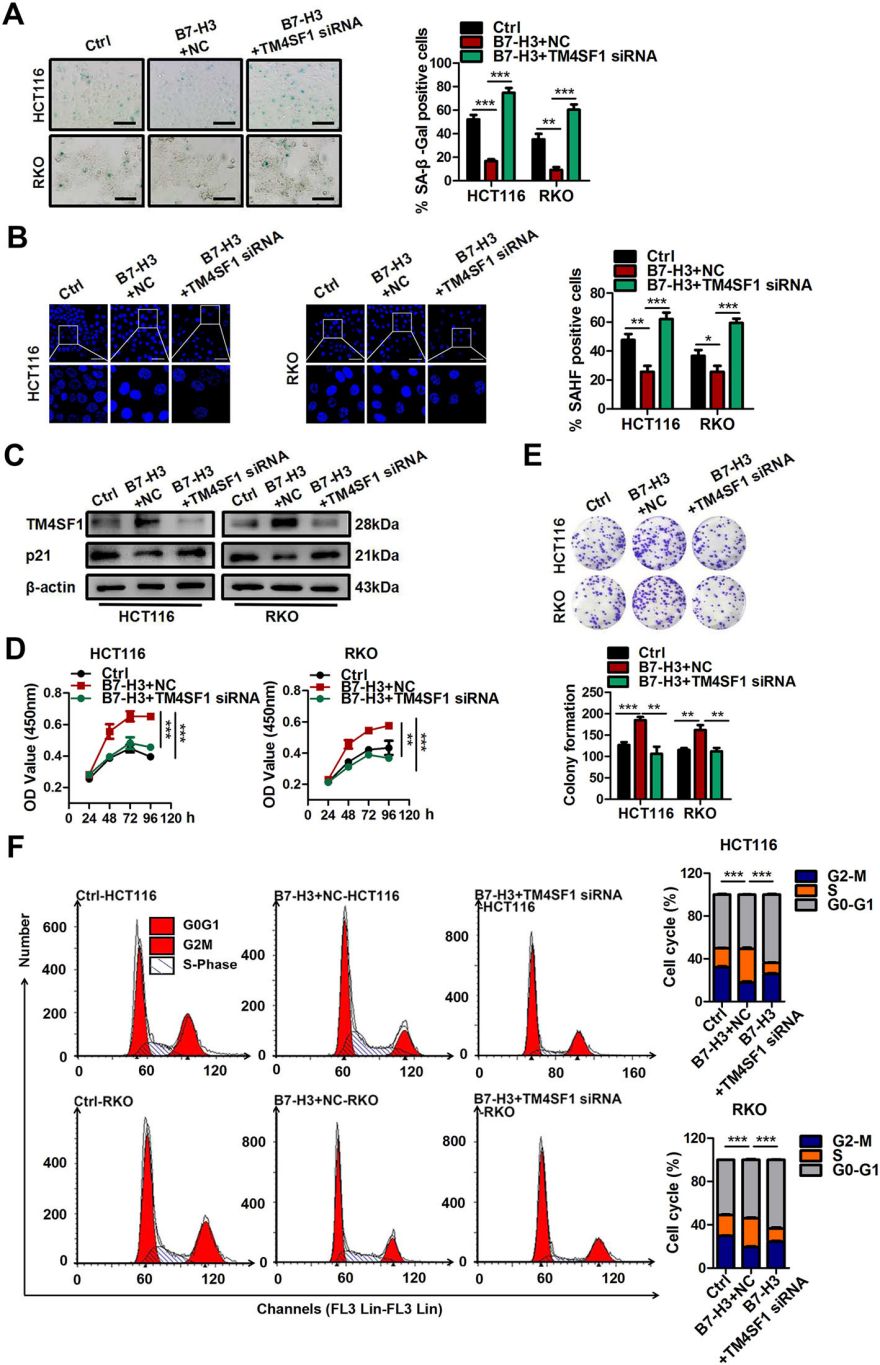
Knockdown of TM4SF1 reverses B7-H3-associated resistance to DOX-induced senescence. **A** SA-β-Gal activity of control cells or B7-H3 CRC cells cotreated with TM4SF1 siRNA and DOX was examined. Scale bar, 100 μm. One representative image from three reproducible experiments is shown. The percentages of SA-β-gal-positive cells are shown in the bar graph. **B** SAHF activity of control cells or B7-H3 CRC cells cotreated with TM4SF1 siRNA and DOX was examined. Scale bar, 50 μm. One representative image from three replicate experiments. **C** Western blot analysis of TM4SF1 and p21 in control cells or B7-H3 CRC cells cotreated with TM4SF1 siRNA and DOX. β-Actin served as a loading control. **D** The cell viability of control cells or B7-H3 CRC cells cotreated with TM4SF1 siRNA and DOX after 24, 36, 48, and 96 h was examined by CCK-8 assays. **E** The colony formation of control cells or B7-H3 CRC cells with TM4SF1 siRNA and DOX cotreatment was examined. One representative image from three reproducible experiments is shown. The number of colonies is shown in the bar graph. **F** Cell cycle analysis by PI staining in control cells or B7-H3 CRC cells with TM4SF1 siRNA and DOX cotreatment was examined through flow cytometry. The data represent the mean ± SEM. **P < 0.01; ***P < 0.001.

The above-described results suggested that TM4SF1 may be a key downstream target molecule of B7-H3 in CRC cells treated with low-dose DOX. Hence, we further investigated whether the effect of B7-H3 on cellular senescence was TM4SF1 dependent. The Western blot results showed that TM4SF1 overexpression upregulated p21 protein levels in low-dose DOX-induced senescent shB7-H3 HCT116 and RKO cells (Fig. 4A). In addition, as shown in Fig. 4B–E, TM4SF1 overexpression partially reversed the effect of B7-H3 knockdown on increased percentages of SA-β-gal- and SAHF-positive cells in CRC cells treated with low-dose DOX (Fig. 4B–E). Moreover, the increase in cell growth arrest and G2/M phase arrest were partially alleviated by TM4SF1 overexpression in shB7-H3 CRC cells treated with low-dose DOX (Fig. 4F–H). Moreover, we filtered and obtained the most effective TM4SF1 siRNA-2 among three commercial siRNAs targeting TM4SF1, as detected by both RT-qPCR and western blot assays (Supplementary Fig. S3A, B). Treatment with TM4SF1 siRNA-2 led to a significant increase in the percentages of SA-β-gal- and SAHF-positive cells (Fig. 5A, B), as well as the protein level of p21 in B7-H3-overexpressing CRC cells treated with low-dose DOX (Fig. 5C). Moreover, TM4SF1 siRNA-2 transfection abolished the decrease in cell growth arrest and G2/M phase arrest in B7-H3-overexpressing CRC cells treated with low-dose DOX (Fig. 5D–F). These results illustrated that B7-H3 suppressed cellular senescence and senescence-like cell growth arrest via TM4SF1.

### B7-H3-mediated promotion of TM4SF1 expression and inhibition of cellular senescence is dependent on the AKT pathway in CRC

**Fig. 6:**
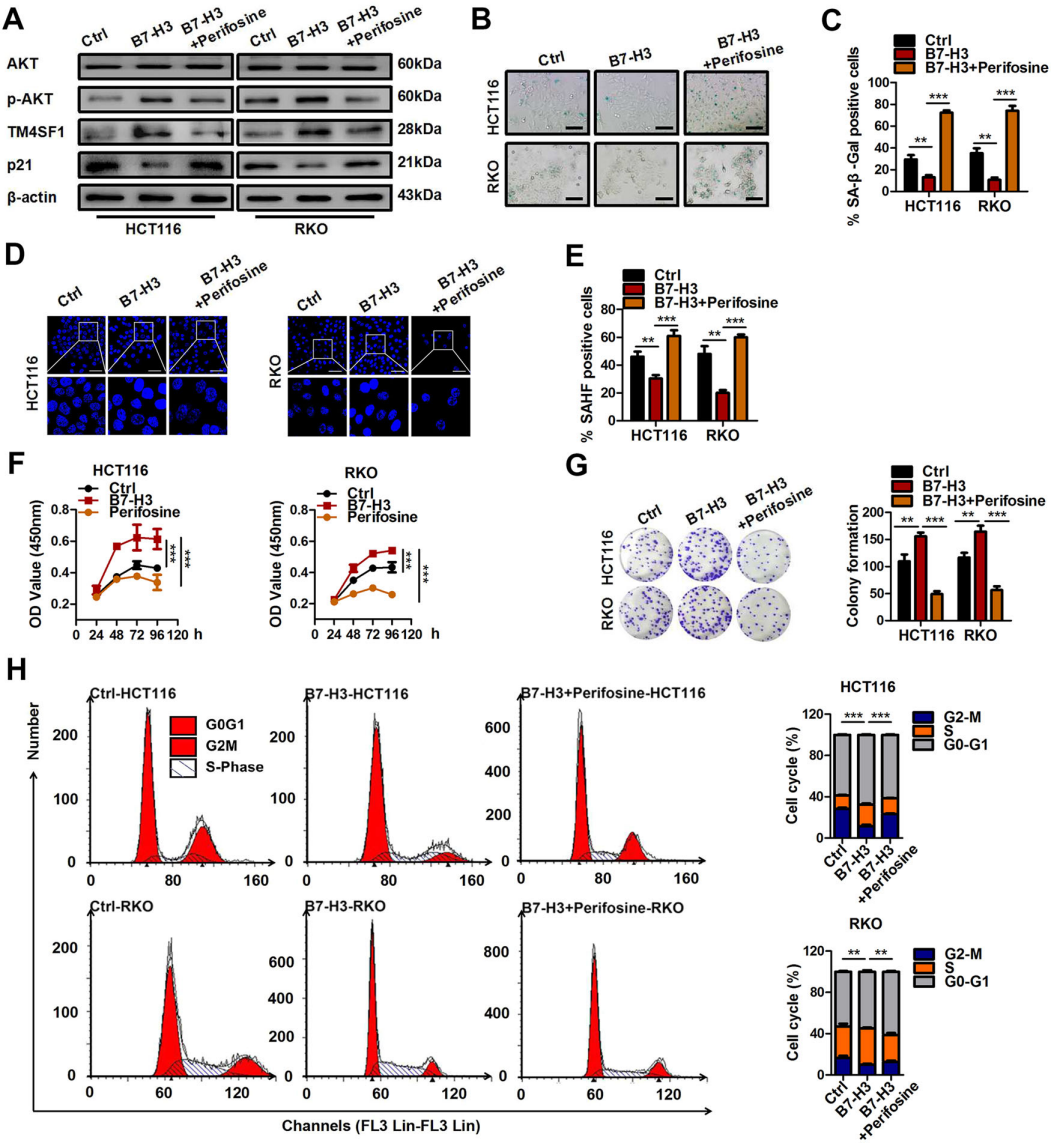
B7-H3 promotes TM4SF1 expression and inhibits cellular senescence dependent on the AKT pathway in CRC. **A** Western blot analysis of AKT, p-AKT, TM4SF1 and p21 in control cells or B7-H3 CRC cells cotreated with perifosine and DOX. β-Actin served as a loading control. **B**, **C** SA-β-Gal activity of control cells or B7-H3 CRC cells cotreated with perifosine and DOX was examined in CRC cells. Scale bar, 100 μm. One representative image from three reproducible experiments is shown. The percentages of SA-β-gal-positive cells are shown in the bar graph. **D**, **E** SAHF activity of control cells or B7-H3 CRC cells cotreated with perifosine and DOX was examined. Scale bar, 50 μm. One representative image from three replicate experiments. **F** The cell viability of control cells or B7-H3 CRC cells cotreated with perifosine and DOX after 24, 36, 48, and 96 h was examined by CCK-8 assays. **G** Colony formation assay in control cells or B7-H3 CRC cells cotreated with perifosine and DOX. One representative image from three reproducible experiments is shown. The number of colonies is shown in the bar graph. **H** Cell cycle analysis by PI staining in control cells or B7-H3 CRC cells with perifosine and DOX cotreatment was examined through flow cytometry. The data represent the mean ± SEM. **P < 0.01; ***P < 0.001.

Our previous studies showed that B7-H3 overexpression activated downstream signaling pathways, such as the AKT, NF-κB, and STAT3 pathways, in CRC cells^27^. In the present study, Kyoto Encyclopedia of Genes and Genomes (KEGG) analysis showed that the AKT pathway was identified as a differentially enriched pathway in B7-H3 knockdown RKO cells treated with low-dose DOX (Supplementary Fig. S4A). Hence, we hypothesized that B7-H3 might promote TM4SF1 expression in low-dose DOX-induced senescent CRC cells via the AKT pathway. As shown in Supplementary Fig. S5B, treatment with the AKT inhibitor perifosine significantly reduced the mRNA level of TM4SF1 in low-dose DOX-induced senescent B7-H3-overexpressing HCT116 and RKO cells, while NF-κB inhibitor (BAY11–7082) or STAT3 inhibitor (cryptotanshinone) treatment had no effect on TM4SF1 mRNA expression. Moreover, perifosine administration obviously reduced the phosphorylation levels of AKT and the protein expression of TM4SF1 in low-dose DOX-induced senescent B7-H3-overexpressing HCT116 and RKO cells (Fig. 6A), suggesting that B7-H3 promoted TM4SF1 expression in an AKT pathway-dependent manner. We then asked whether the AKT pathway was attributed to B7-H3-regulated cellular senescence in CRC cells. Perifosine treatment led to a significant increase in the percentages of SA-β-gal- and SAHF-positive cells (Fig. 6B–E), as well as the protein level of p21, in B7-H3-overexpressing CRC cells treated with low-dose DOX (Fig. 6A). Furthermore, perifosine administration abolished the decrease in cell growth arrest and G2/M phase arrest in B7-H3-overexpressing CRC cells treated with low-dose DOX (Fig. 6F–H).

### SIRT1 is required for B7-H3/TM4SF1 axis-regulated cellular senescence

**Fig. 7:**
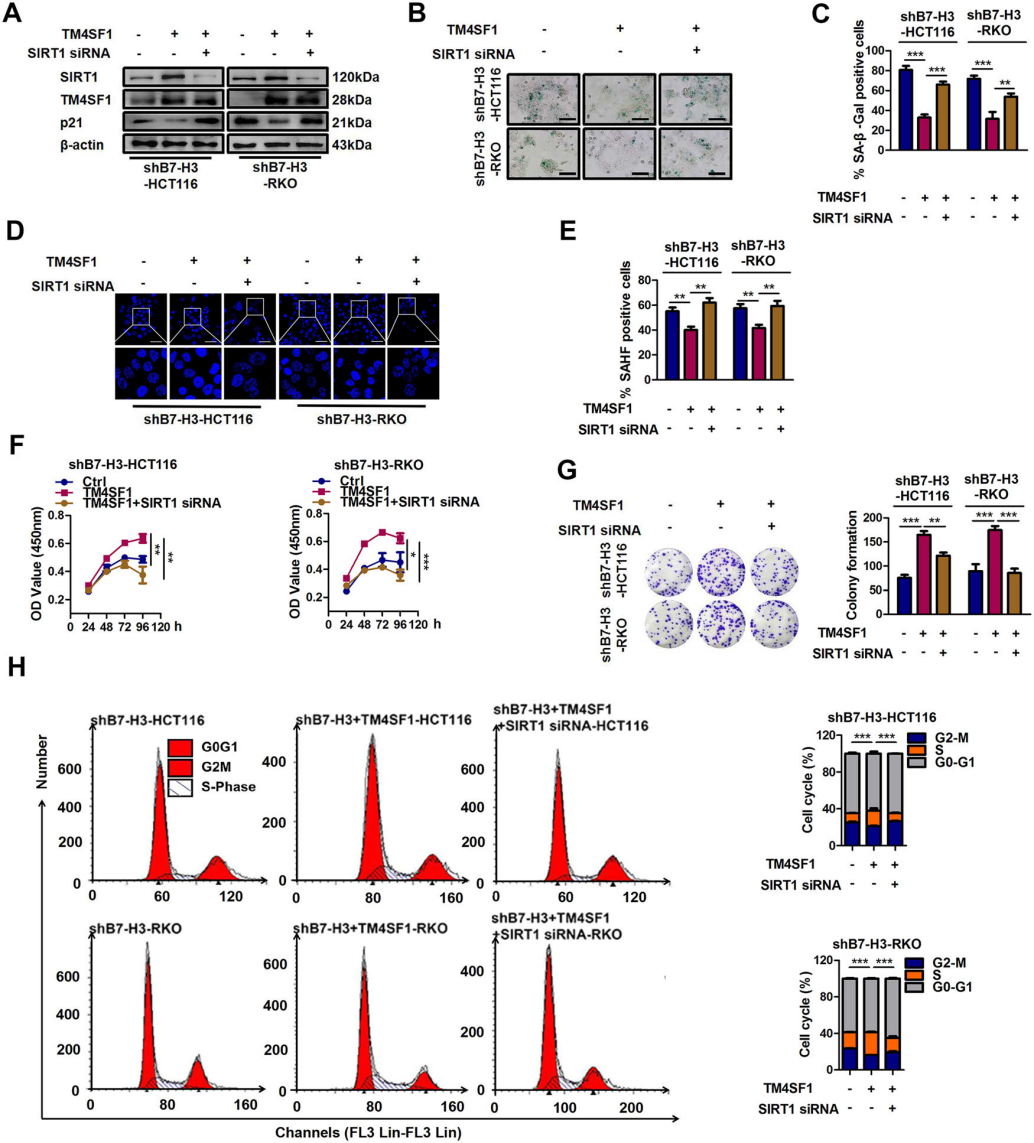
SIRT1 is required for the resistance to B7-H3-mediated senescence. **A** Western blot analysis of SIRT1, TM4SF1, and p21 in DOX-treated shB7-H3 CRC cells cotreated with TM4SF1 overexpression vectors and SIRT1 siRNA. β-Actin served as a loading control. **B**, **C** SA-β-Gal activity of DOX-treated shB7-H3 CRC cells with TM4SF1 overexpression vectors and SIRT1 siRNA cotreatment was examined. Scale bar, 100 μm. One representative image from three reproducible experiments is shown. The percentages of SA-β-gal-positive cells are shown in the bar graph. **D**, **E** SAHF activity of DOX-treated shB7-H3 CRC cells with TM4SF1 overexpression vectors and SIRT1 siRNA cotreatment was examined. Scale bar, 50 μm. One representative image from three replicate experiments. **F** The cell viability of control cells or DOX-treated shB7-H3 CRC cells cotreated with TM4SF1 overexpression vectors and SIRT1 siRNA after 24, 36, 48, and 96 h was examined by CCK-8 assays. **G** The colony formation of control cells or DOX-treated shB7-H3 CRC cells with TM4SF1 overexpression vectors and SIRT1 siRNA cotreatment was examined. One representative image from three reproducible experiments is shown. The number of colonies is shown in the bar graph. **H** Cell cycle analysis by PI staining in control cells or DOX-treated shB7-H3 CRC cells with TM4SF1 overexpression vectors and SIRT1 siRNA cotreatment was examined through flow cytometry. The data represent the means ± SEM. *P < 0.05; **P < 0.01; ***P < 0.001.

Sirtuin 1 (SIRT1), a key member of the deacetylase Sirtuin family, has been shown to play vital roles in modulating cellular senescence and aging^34^. In addition, TM4SF1 was involved in apoptosis, the cell cycle, and ROS metabolism of bladder cancer cells through the PPARγ-SIRT1 feedback loop^35^. Thus, it becomes indispensable to find the relationship between SIRT1 and the B7-H3/TM4SF1 axis in low-dose DOX-induced cellular senescence. The western blot results showed that B7-H3 knockdown decreased SIRT1 protein expression, while TM4SF1 overexpression reversed the effect of B7-H3 knockdown on SIRT1 expression in low-dose DOX-induced senescent HCT116 and RKO cells (Supplementary Fig. S5A). Moreover, B7-H3 overexpression increased SIRT1 protein expression, while TM4SF1 knockdown abolished the effect of B7-H3 overexpression on SIRT1 expression in low-dose DOX-induced senescent CRC cells (Supplementary Fig. S5B). These results indicated that the B7-H3/TM4SF1 axis was able to regulate SIRT1 expression in low-dose DOX-induced senescent CRC cells. To further investigate the roles of SIRT1 in B7-H3/TM4SF1 axis-regulated cellular senescence, we used a commercial SIRT1 siRNA and a TM4SF1 overexpression vector to cotransfect HCT116 and RKO cells. The results showed that TM4SF1 overexpression increased SIRT1 protein expression, while SIRT1 knockdown abolished the effect of TM4SF1 overexpression on SIRT1 expression in low-dose DOX-induced senescent shB7-H3 CRC cells (Fig. 7A). Moreover, SIRT1 knockdown reversed the effect of TM4SF1 overexpression on the percentages of SA-β-gal- and SAHF-positive cells (Fig. 7B–E), as well as the protein level of p21 in B7-H3 knockdown CRC cells treated with low-dose DOX (Fig. 7A). In addition, SIRT1 knockdown abolished the decrease in cell growth arrest and G2/M phase arrest induced by TM4SF1 overexpression in B7-H3 knockdown CRC cells treated with low-dose DOX (Fig. 7F–H). The above results indicate that the B7-H3/TM4SF1 axis modulates low-dose DOX-induced cellular senescence via SIRT1.

### B7-H3 inhibited DOX-induced cellular senescence in vivo

To verify the role of B7-H3 in DOX-induced cellular senescence in vivo, we first established a low-dose DOX-induced cell senescence model in nude mice. We found that low-dose DOX treatment (4 mg/kg) significantly reduced the sizes and weight of tumors (Supplementary Fig. S6A–C). More importantly, treatment with low-dose DOX increased the percentage of SA-β-gal staining and the expression of p21 in tumors (Supplementary Fig. S6D, E). These results suggested that treatment with low-dose DOX induced cellular senescence in vivo.

**Fig. 8:**
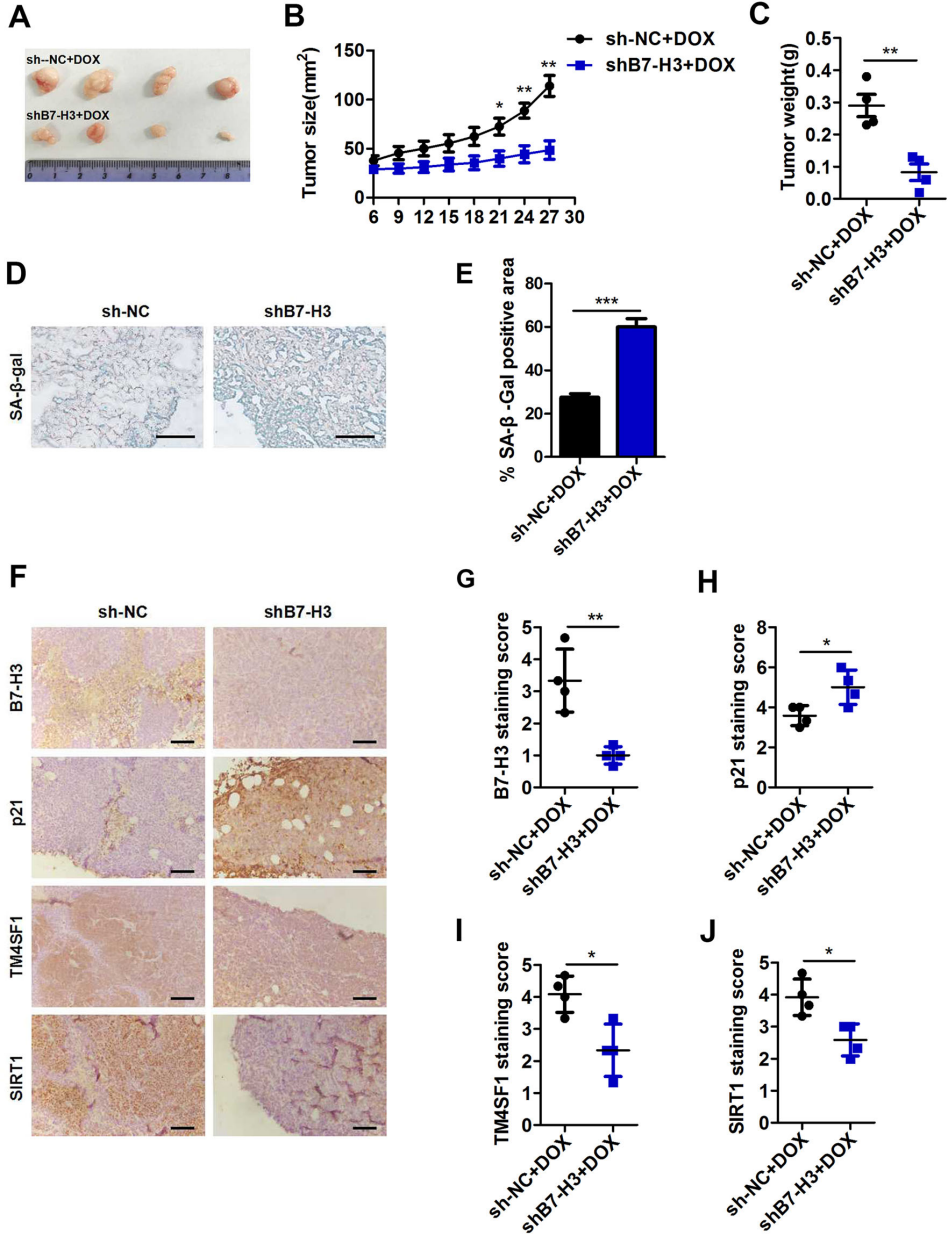
B7-H3 inhibited DOX-induced cellular senescence in vivo. **A** Images of the subcutaneous tumors formed by sh-NC-HCT116 and shB7-H3-HCT116 cells treated with DOX (4 mg/kg). *N* = 4. **B**, **C** Quantification of the size and weight of subcutaneous tumors formed by sh-NC-HCT116 and shB7-H3-HCT116 cells treated with DOX. **D**, **E** Representative Images and SA-β-Gal activity of the subcutaneous tumors formed by sh-NC-HCT116 and shB7-H3-HCT116 cells treated with DOX. *N* = 4. **F** Representative images of IHC for B7-H3, p21, TM4SF1 and SIRT1 in subcutaneous tumors formed by sh-NC-HCT116 and shB7-H3-HCT116 cells treated with DOX. *N* = 4. **G**–**J** B7-H3, p21, TM4SF1, and SIRT1 protein expression based on their IHC staining index results in subcutaneous sh-NC-HCT116 and shB7-H3-HCT116 tumors treated with DOX. The data represent the means ± SEM. *P < 0.05; **P < 0.01; ***P < 0.001.

B7-H3 knockdown significantly decreased HCT116 tumor growth after treatment with low-dose DOX, evidenced by the tumor sizes, images, and weight (Fig. 8A–C). B7-H3 overexpression had the opposite effect (Supplementary Fig. S7A–C). The percentage of SA-β-gal staining and the expression of p21 were obviously increased in shB7-H3 HCT116 tumors treated with low-dose DOX (Fig. 8D–H). Conversely, decreased percentage of SA-β-gal staining and p21 expression was observed in B7-H3 HCT116 tumors treated with low-dose DOX (Supplementary Fig. S7D–H). Moreover, B7-H3 knockdown significantly decreased, while B7-H3 overexpression markedly increased the expression of TM4SF1 and SIRT1 in HCT116 tumors treated with low-dose DOX (Fig. 8I, J and Supplementary Fig. S7I–J). Taken together, we illustrated that B7-H3 could inhibit DOX-induced cellular senescence of CRC cells in vivo by regulating TM4ST1/SIRT1 expression.

## Discussion

Cellular senescence is considered as a kind of stress response induced by a variety of intrinsic and extrinsic insults^36^. Nowadays, cellular senescence, especially therapy-induced senescence (TIS), has been recognized as a new weapon for cancer therapy^37^. Hence, increasing evidence has indicated the underlying molecular mechanisms of this process in cancer cells. For instance, EZH2 mediates lidamycin-induced cellular senescence in a p21-dependent manner in CRC cells^38^. AP4, the chromatin remodeling enzyme, downregulates p16 and p21 to suppress senescence^39^. Zhang and coworkers found that inhibition of TAZ contributes to radiation-induced senescence and growth arrest in glioma cells^40^. In this study, we induced cellular senescence in CRC cells by a low dose of DOX and demonstrated that B7-H3 knockdown promoted, while B7-H3 overexpression inhibited, cellular senescence induced by a low dose of DOX. Importantly, B7-H3 knockdown dramatically enhanced the growth arrest of CRC cells after low-dose DOX treatment, but the overexpression of B7-H3 had the opposite effect. In addition, B7-H3 mediated cellular senescence induced by a low dose of DOX in vivo. These results suggest that B7-H3 is an important suppressor of low-dose DOX-induced senescence in CRC. Apart from therapy-induced senescence, replicative senescence, oncogene-induced senescence, and PTEN-loss induced cellular senescence also exert important role in tumor progression and therapy^41,42^. At the current stage we could not clarify the precise functions of B7-H3 in regulating replicative senescence, oncogene-induced senescence, and PTEN-loss induced cellular senescence in CRC. Further investigations are required to answer this question.

Recent evidence indicated that cellular senescence acts as a double-edged sword during tumorigenesis because it promotes tumorigenesis mainly through the SASP apart from its tumor-suppressing functions^11^. Thus far, eliminating senescent cells or suppressing SASP has received wide attention. Athena et al. showed that PTBP1 depletion prevented the protumorigenic effects of SASP and inhibited inflammation-driven cancer^43^. Apigenin mediated suppression of the SASP and reduced the aggressive phenotype of human breast cancer cells^44^. It has been reported that B7-H3 is involved in the production of cytokines such as IL-6, IL-8, and VEGF in cancers^27,45,46^. Therefore, we surmised that B7-H3 might play important roles in regulating the SASP in senescent CRC cells induced by a low dose of DOX. Unfortunately, we did not investigate the effect of B7-H3 on SASP in the present study. It would be interesting to further elaborate the functions of B7-H3 in modulating SASP in CRC.

The most widely studied pathways regulating cellular senescence are p53/p21^cip1^ and/or p16^INK4A^/Rb tumor suppressor pathways^47–49^. DNA damage and/or the DNA-damage response (DDR) are seemingly the two crucial parts controlling these two pathways^50,51^. It has been reported that p53 regulates the expression of a great deal of target genes involved in senescence^52^. Herein, we observed that both p53 and p21 protein expression were increased in HCT116 and RKO cells after treatment with a low dose of DOX. However, B7-H3 alteration regulated p21 expression but did not affect the expression of p53 in low-dose DOX-induced senescent CRC cells. These results indicated that B7-H3 regulated p21 expression and low-dose DOX-induced cellular senescence in a p53-independent manner. In line with our results, many novel regulators, such as ARF, TRIB2, and SETD8, are involved in p53-independent cellular senescence^16,53,54^.

To extensively explore the mechanisms responsible for B7-H3-mediated regulation of cellular senescence in CRC cells, we performed RNA-seq analysis to identify the DEGs in shB7-H3 RKO cells treated with low-dose DOX and found that TM4SF1 was one of the highest rated DEGs. TM4SF1, a member of the TM4SF protein family, is identified as an oncogene and regulates proliferation, metastasis, and invasion in various cancers, including CRC^55^. Moreover, it has been reported that TM4SF1 is one of the top 10 upregulated genes and can act as marker of poor prognosis in CRC^56^. In addition, there is evidence that TM4SF1 is also an important regulator of senescence^57,58^. The expression of TM4SF1 was increased in senescent human mesenchymal stem cells, which seemed to affect cellular senescence^57^. Moreover, TM4SF1 regulated endothelial cell functions, including filopodia formation, cell mobility, cytokinesis, cellular senescence, and tumor angiogenesis^58^. In the current work, we verified both the mRNA and protein expression of TM4SF1 in low-dose DOX-induced senescent CRC cells, which was significantly downregulated in B7-H3 knockdown cells but upregulated in B7-H3-overexpressing cells. Furthermore, TM4SF1 expression was positively correlated with B7-H3 expression in human CRC tissues. More importantly, silencing of TM4SF1 obviously abated B7-H3-mediated resistance to cell senescence, while the overexpression of TM4SF1 substantially alleviated the degree of senescence in B7-H3 knockdown CRC cells. Thus, we have shown that B7-H3 enhances the resistance to low-dose DOX-induced senescence of CRC cells via TM4SF1.

AKT is known to be one of the important central nodes in different signaling pathways during tumorigenesis^59^. Frequent hyperactivation of AKT kinases has widely been found in various human solid tumors. Inhibiting the AKT pathway is closely related to changing several biological characteristics in cancers, such as cell proliferation, cell cycle distribution, and cellular senescence^60,61^. Hirose and coworkers showed that AKT activation could suppress temozolomide-induced mitotic catastrophe and cellular senescence in glioblastoma cells^62^. In the present study, KEGG pathway enrichment analysis indicated that DEGs in B7-H3 knockdown RKO cells treated with low-dose DOX were clearly enriched in the AKT pathway. Previous studies have shown that TM4SF1 is well known for its oncogenic functions in human cancers via the AKT pathway^33,63^. Additionally, we previously demonstrated that B7-H3 overexpression could activate the AKT pathway in CRC cells^27^. Therefore, we hypothesized that B7-H3 might promote TM4SF1 expression in low-dose DOX-induced senescent CRC cells via the AKT pathway. As expected, our Western blot assay indicated that perifosine, an AKT inhibitor, markedly reduced the phosphorylation levels of AKT and the expression of TM4SF1 in low-dose DOX-induced senescent B7-H3-overexpressing CRC cells, providing evidence that B7-H3 modulated TM4SF1 expression in senescent CRC cells via the AKT signaling pathway. Importantly, we observed that perifosine treatment led to a significant decrease in B7-H3-mediated resistance to cellular senescence. This suggests that B7-H3 is involved in low-dose DOX-induced senescence of CRC cells via the AKT/TM4SF1 pathway.

SIRT1 is categorized as a class III histone deacetylase and plays a role in a wide range of cellular functions, such as metabolism, differentiation, and senescence^64–66^. Importantly, SIRT1 overexpression has been discovered in many cancers, including CRC^67^. Moreover, the expression level of TM4SF1 obviously influenced the expression of SIRT1 in senescent models, as reported previously^35^. It has been confirmed that p21, an important cellular senescence marker, can be suppressed by SIRT1 in a p53-dependent and independent manner^68,69^. These findings led us to postulate that SIRT1 is of great importance in B7-H3-mediated resistance to cellular senescence through the AKT/TM4SF1 axis. Consistent with the change in TM4SF1 expression, SIRT1 expression was reduced in B7-H3 knockdown cells but increased in B7-H3-overexpressing cells. Moreover, TM4SF1 overexpression reversed the decrease in SIRT1 protein expression caused by B7-H3 knockdown in low-dose DOX-induced senescent CRC cells. Consistently, TM4SF1 knockdown abolished the effect of B7-H3 overexpression on SIRT1 expression in low-dose DOX-induced senescent CRC cells. More importantly, SIRT1 knockdown abolished B7-H3/TM4SF1 axis-mediated resistance to cell senescence. These results firmly indicate that B7-H3 confers resistance to low-dose DOX-induced senescence mainly through the AKT/TM4SF1/SIRT1 pathway. Although our results indicated that B7-H3/AKT/TM4SF1/SIRT1 axis is able to regulate p21 expression in low-dose DOX-induced senescent CRC cells, the precise way of this axis to p21 expression is still unclear.

Nowadays, several blocking antibodies against B7-H3 antibodies have been developed and used for the therapy of solid tumors. 8H9, a monoclonal antibody specific for 4Ig-B7-H3, has a potent effect for patients with B7-H3 positive tumors in radioimmunotherapy^70^. Besides, humanized monoclonal antibodies targeting B7-H3, enoblituzumab, are under clinical investigation for therapeutic use in human prostate cancer^71^. Given our results that B7-H3 played important roles in low-dose DOX-induced senescence of CRC cells in vitro and in vivo, we speculated that combination B7-H3 blockade therapy and TIS therapy based on low-dose DOX would become a promising strategy for CRC patients.

Our findings, for the first time, indicate that the high expression of B7-H3 exacerbates the resistance to low-dose DOX-induced senescence through the AKT/TM4SF1/SIRT1 pathway. B7-H3 may act not only as a biomarker for predicting the response to low-dose DOX-induced senescence before it becomes clinically apparent but also as a target for potential paths for the development clinical interventions, which may improve the efficiency of TIS in the future.

## Supplementary information

Supplementary tables and figure legends

Figure S1

Figure S2

Figure S3

Figure S4

Figure S5

Figure S6

Figure S7
