# Supplementary material for: B7-H3 suppresses doxorubicin-induced senescence-like growth arrest in colorectal cancer through the AKT/TM4SF1/SIRT1 pathway
Source: Cell Death Dis. 2021 May 6;12(5):453. doi: 10.1038/s41419-021-03736-2 (PMC8102521; doi:10.1038/s41419-021-03736-2)
Supplement: Supplementary file 1 — Supplementary tables and figure legends [file 41419_2021_3736_MOESM1_ESM.doc]

**Supplementary Table 1. Clinical characteristics of patients**

| CRC patients | Number |
| --- | --- |
| NO. of patients | 54 |
| Gender |  |
| Male | 33 |
| Female | 21 |
| Age (years) |  |
| Mean | 60.63 |
| Range | 26-79 |
| Tumor location |  |
| Colon | 34 |
| Rectun | 20 |
| TNM stage |  |
| I-II | 26 |
| III-IV | 28 |

**Supplementary Table 2. Antibodies for Western b**olt in this study

| Antibody Name | Details |
| --- | --- |
| AKT | CST, #4691, 1:1000 |
| p-AKT | CST, #4060, 1:2000 |
| STAT3 | CST, #9139, 1:1000 |
| p-STAT3 | CST, #9145, 1:2000 |
| NF-κB p65 | Beyotime, #AN365, 1:500 |
| p-NF-κB-p65 | Beyotime, #AN371, 1:1000 |
| human B7-H3 | R&D Systems, #AF1027, 1:250 |
| β-actin | Immunoway Biotechnology, #YM3028, 1:5000 |
| TM4SF1 | Abcam, #ab113504, 1:1000 |
| SIRT1 | Beyotime, #AF0282, 1:1000 |
| p21 | Beyotime, #AP021, 1:500 |
| p53 | Beyotime, #AF0255, 1:1000 |

**Supplementary Table 3**. Primers for RT-qPCR in this study

| Primer Name | Primer Sequence(5’-3’） |
| --- | --- |
| hsa-TM4SF1-Forward | TGCATCGGACATTCTCTGGTG |
| hsa-TM4SF1-Reverse | GTTCCAGCCCAATGAAGACAA |
| hsa-B7-H3-Forward | ACAGGGCAGCCTATGACATT |
| hsa-B7-H3-Reverse | CTGCATTCTCCTCCTCACAG |
| hsa-β-actin-Forward | AAGGAGCCCCACGAGAAAAAT |
| hsa-β-actin-Reverse | ACCGAACTTGCATTGATTCCAG |

**Supplementary Table 4. DEGs with logarithm of fold change (|logFC|) > 10 in shB7-H3 RKO cells treated** with low dose DOX were identified in RNA-seq analysis

| **geneID** | **GeneSymbol** | **foldChange** | **log2FoldChange** | **Regulation** | **pval** |
| --- | --- | --- | --- | --- | --- |
| ENSG00000159167 | STC1 | 0.0001091 | -13.16255 | Down | 1.42E-20 |
| ENSG00000019991 | HGF | 0.0003706 | -11.39767 | Down | 9.08E-16 |
| ENSG00000169908 | TM4SF1 | 0.0001564 | -12.64228 | Down | 1.02E-15 |
| ENSG00000123689 | G0S2 | 0.0001739 | -12.48985 | Down | 3.18E-15 |
| ENSG00000197769 | MAP1LC3C | 0.0001995 | -12.29117 | Down | 1.41E-14 |
| ENSG00000117152 | RGS4 | 0.0005388 | -10.85798 | Down | 1.18E-15 |
| ENSG00000229807 | XIST | 0.0005938 | -10.71785 | Down | 3.15E-15 |
| ENSG00000038427 | VCAN | 1563.3333 | 10.61041 | Ups | 2.83E-13 |
| ENSG00000163565 | IFI16 | 0.0009741 | -10.00366 | Down | 5.19E-13 |
| ENSG00000153956 | CACNA2D1 | 0.0005789 | -10.75447 | Down | 2.63E-12 |
| ENSG00000225972 | MTND1P23 | 0.000956 | -10.03067 | Down | 1.83E-11 |
| ENSG00000140379 | BCL2A1 | 0.0005659 | -10.78709 | Down | 6.98E-10 |
| ENSG00000168461 | RAB31 | 1355 | 10.404077 | Ups | 8.24E-09 |
| ENSG00000002587 | HS3ST1 | 1343 | 10.391244 | Ups | 8.93E-09 |
| ENSG00000114631 | PODXL2 | 0.0007519 | -10.37721 | Down | 9.74E-09 |
| ENSG00000022556 | NLRP2 | 1223 | 10.256209 | Ups | 2.05E-08 |
| ENSG00000186868 | MAPT | 1170 | 10.192293 | Ups | 3.01E-08 |
| ENSG00000076706 | MCAM | 1154 | 10.172428 | Ups | 3.39E-08 |
| ENSG00000205189 | ZBTB10 | 1152 | 10.169925 | Ups | 3.44E-08 |
| ENSG00000254004 | ZNF260 | 0.0009009 | -10.11634 | Down | 4.73E-08 |
| ENSG00000159228 | CBR1 | 0.0009381 | -10.05799 | Down | 6.67E-08 |
| ENSG00000233491 | AC010091.1 | 0.0009737 | -10.00422 | Down | 9.11E-08 |

**Supplementary Figure legends**

**Supplementary Fig. S1. B7-H3 expression level in stable CRC cell lines. A** The mRNA expression of B7-H3 in CRC stable cell lines with B7-H3 inhibition (shB7-H3) or their control cell lines (sh-NC). **B** The mRNA expression of B7-H3 in CRC stable cell lines with B7-H3 overexpression (B7-H3) or their control cell lines (EV).

**Supplementary Fig. S2. The expression of B7-H3 and TM4SF1 was associated with TNM stages and lymph node metastasis in tissue samples of CRC patients. A** B7-H3 protein expression based on the staining index of CRC specimens and matched normal tissues. **B** B7-H3 protein expression based on their staining index in CRC specimens at different clinical stages. **C, D** B7-H3 and TM4SF1 protein expression based on their staining index in CRC specimens with or without lymph node metastasis. The data represent the means ± SEM. **P<0.01; ***P<0.001.

**Supplementary Fig. S3. TM4SF1 siRNAs decreased the expression of TM$SF1 in CRC cell lines. A** The mRNA expression of TM4SF1 in CRC cells treated with three TM4SF1 siRNAs . **B** The protein expression of TM4SF1 in CRC cells treated with three TM4SF1 siRNAs.

**Supplementary Fig. S4. The signaling pathways associated with DOX-induced senescence in CRC cell lines. A, B** RNA sequencing (RNA-seq) analysis of sh-NC cells or shB7-H3 RKO cells with DOX treatment shows different changes associated with DOX-induced senescence in various signaling pathways. **C** Relative mRNA level of TM4SF1 in B7-H3 CRC cells after treatment with perifosine, BAY11-7082 or cryptotanshinone. The data represent the means ± SEM. NS, no significant difference. *P<0.05; ***P<0.001.

**Supplementary Fig. S5. B7-H3/TM4SF1 axis regulated the protein expression of SIRT1 in CRC cell lines. A** The protein expression of TM4SF1 and SIRT1 in control cells and shB7-H3 cells treated with or without the overexpression of TM4SF1. **B** The protein expression of TM4SF1 and SIRT1 in control cells and shB7-H3 cells treated with or without the knowdown of TM4SF1.

**Supplementary Fig. S6. DOX induced cellular senescence in vivo. A** Images of the subcutaneous tumors formed by HCT116 cells treated with or without DOX (4 mg/kg). N = 5. **B, C** Quantification of the size and weight of subcutaneous tumors formed by HCT116 cells treated with or without DOX. **D** Representative Images and SA-β-Gal activity of the subcutaneous tumors formed by HCT116 cells treated with or without DOX. N = 5. **E** Representative images of IHC for p21 and p21 protein expression based on their IHC staining index results in subcutaneous HCT116 tumors treated with or without DOX. N = 5. The data represent the means ± SEM. *P<0.05; **P<0.01.

**Supplementary Fig. S7. B7-H3 inhibited DOX-induced cellular senescence in vivo. A** Images of the subcutaneous tumors formed by EV-HCT116 and B7-H3-HCT116 cells treated with DOX (4 mg/kg). N = 4. **B, C** Quantification of the size and weight of subcutaneous tumors formed by EV-HCT116 and B7-H3-HCT116 cells treated with DOX. **D, E** Representative Images and SA-β-Gal activity of the subcutaneous tumors formed by EV-HCT116 and B7-H3-HCT116 cells treated with DOX. N = 4. **F** Representative images of IHC for B7-H3 , p21, TM4SF1 and SIRT1 in subcutaneous tumors formed by EV-HCT116 and B7-H3-HCT116 cells treated with DOX. N = 4. (G-J) B7-H3 , p21, TM4SF1 and SIRT1 protein expression based on their IHC staining index results in subcutaneous EV-HCT116 and B7-H3-HCT116 tumors treated with DOX. The data represent the means ± SEM. *P<0.05; **P<0.01.
