# Supplementary figures and images for: B7-H3 suppresses doxorubicin-induced senescence-like growth arrest in colorectal cancer through the AKT/TM4SF1/SIRT1 pathway
Source: Cell Death Dis. 2021 May 6;12(5):453. doi: 10.1038/s41419-021-03736-2 (PMC8102521; doi:10.1038/s41419-021-03736-2)

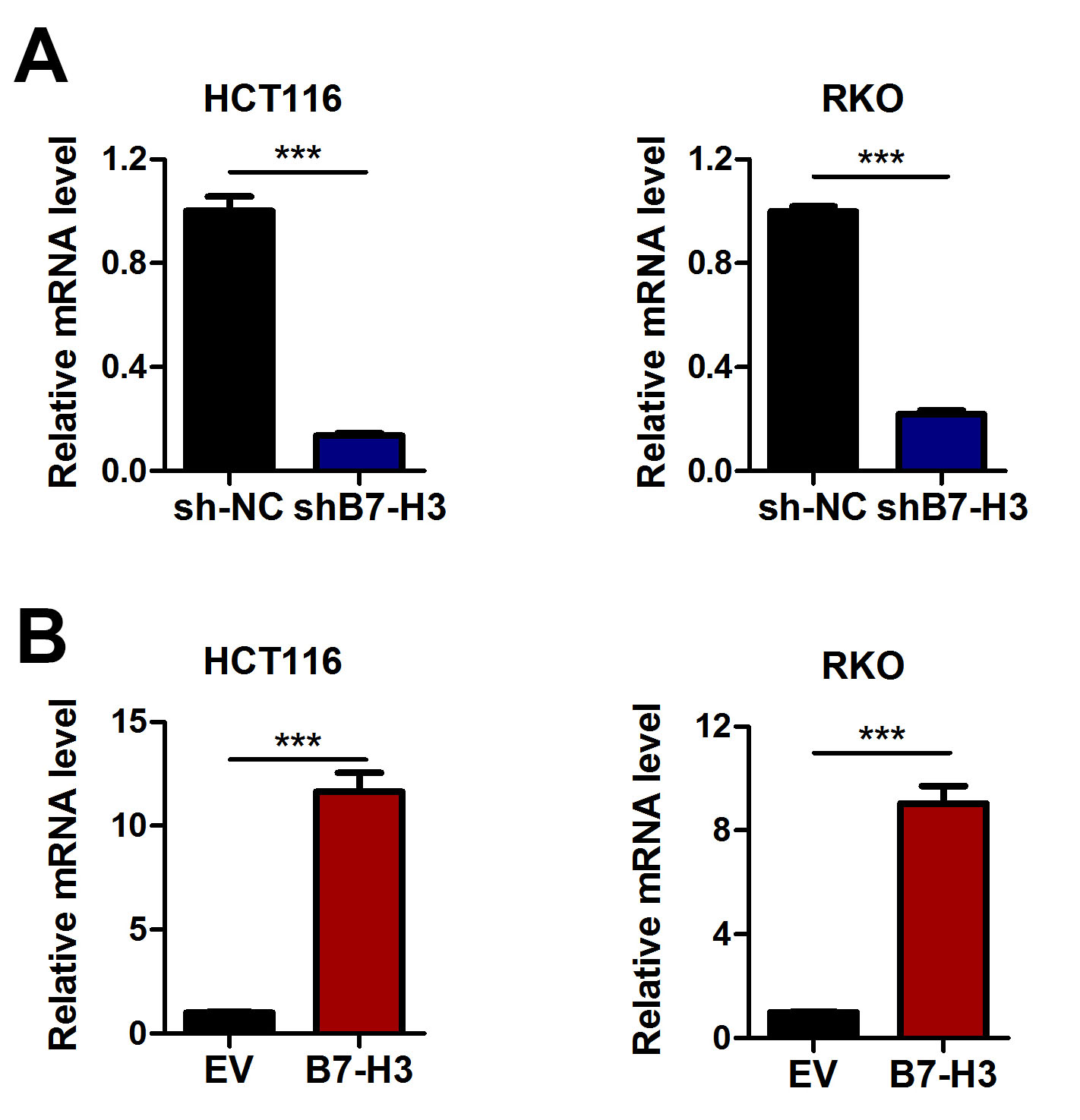

Supplement: Supplementary file 2 — Figure S1 [file 41419_2021_3736_MOESM2_ESM.jpg]

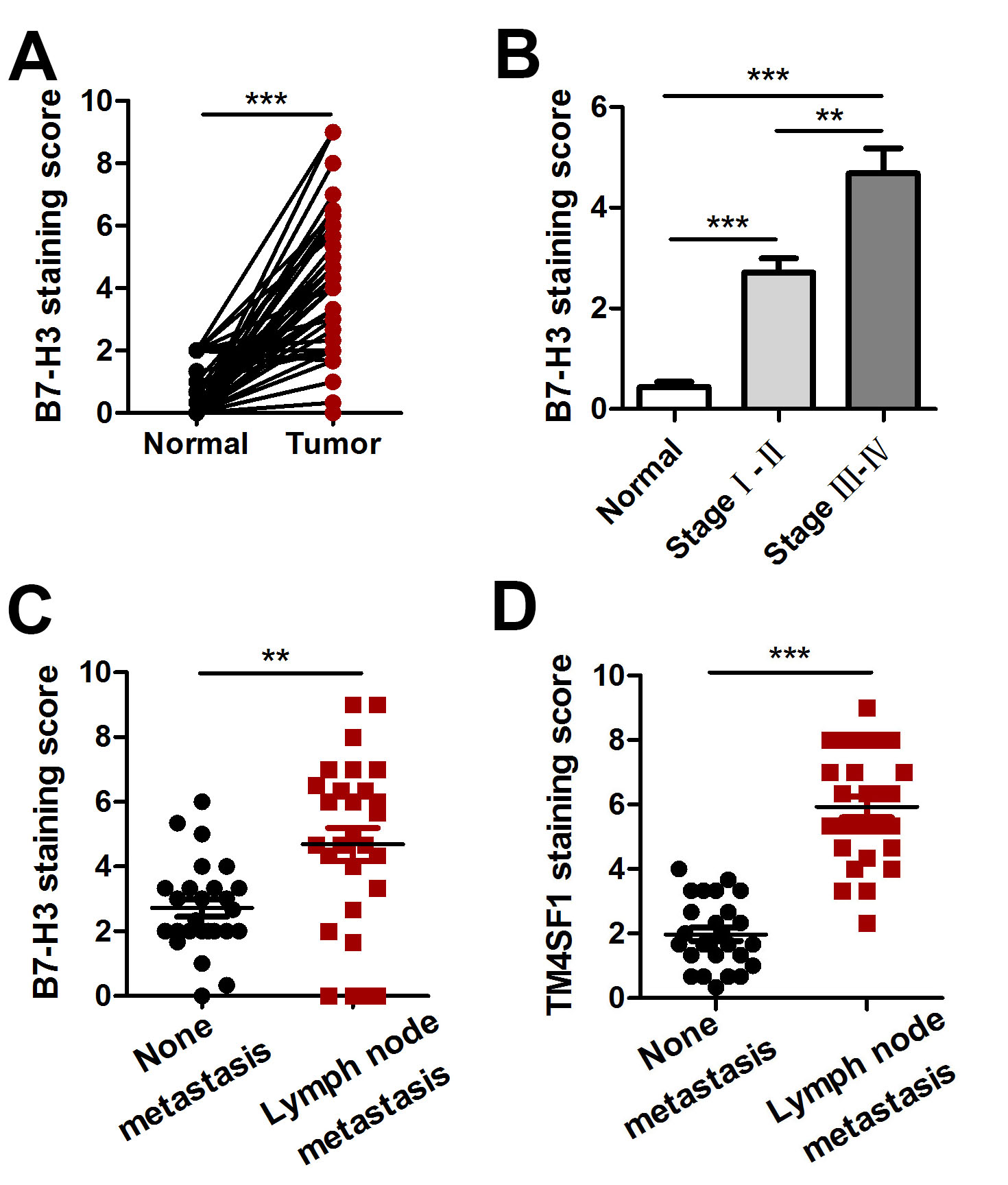

Supplement: Supplementary file 3 — Figure S2 [file 41419_2021_3736_MOESM3_ESM.jpg]

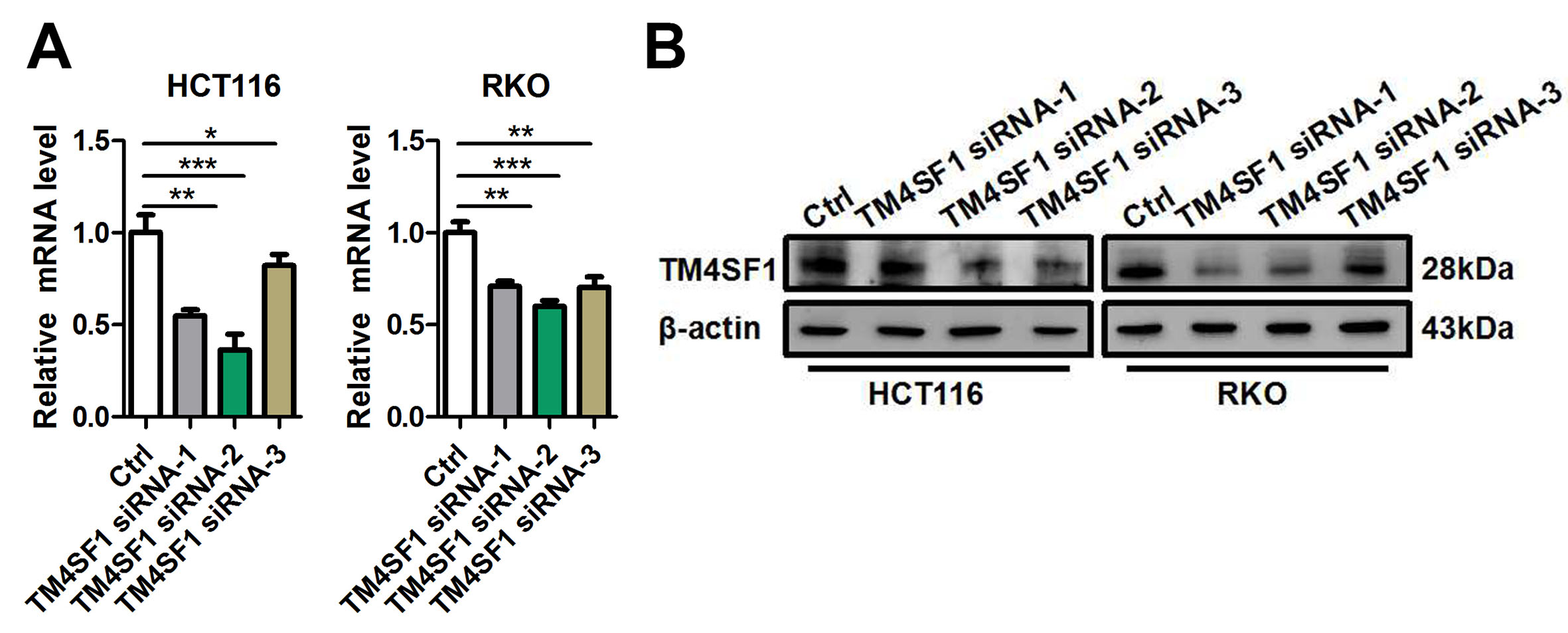

Supplement: Supplementary file 4 — Figure S3 [file 41419_2021_3736_MOESM4_ESM.jpg]

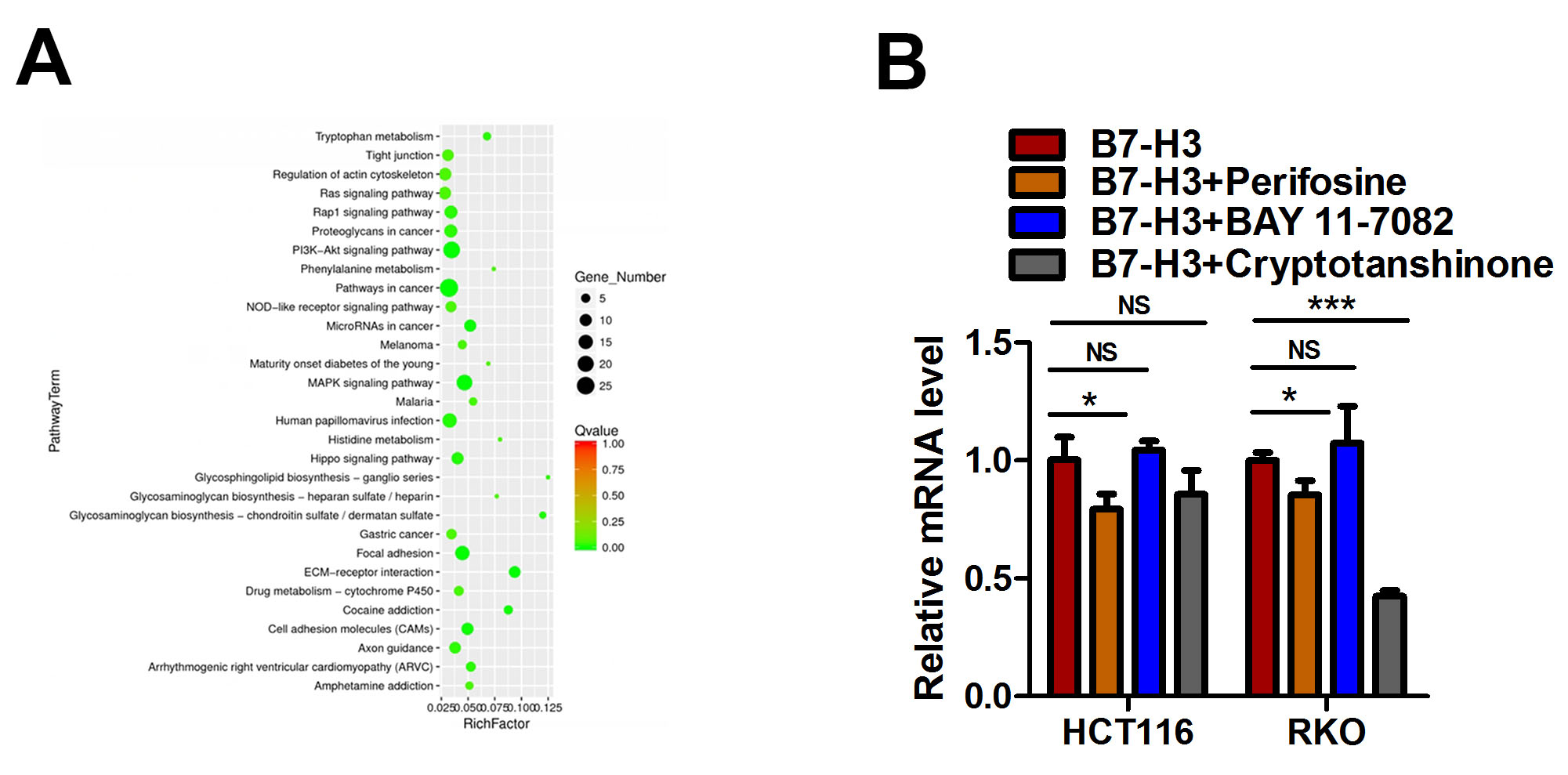

Supplement: Supplementary file 5 — Figure S4 [file 41419_2021_3736_MOESM5_ESM.jpg]

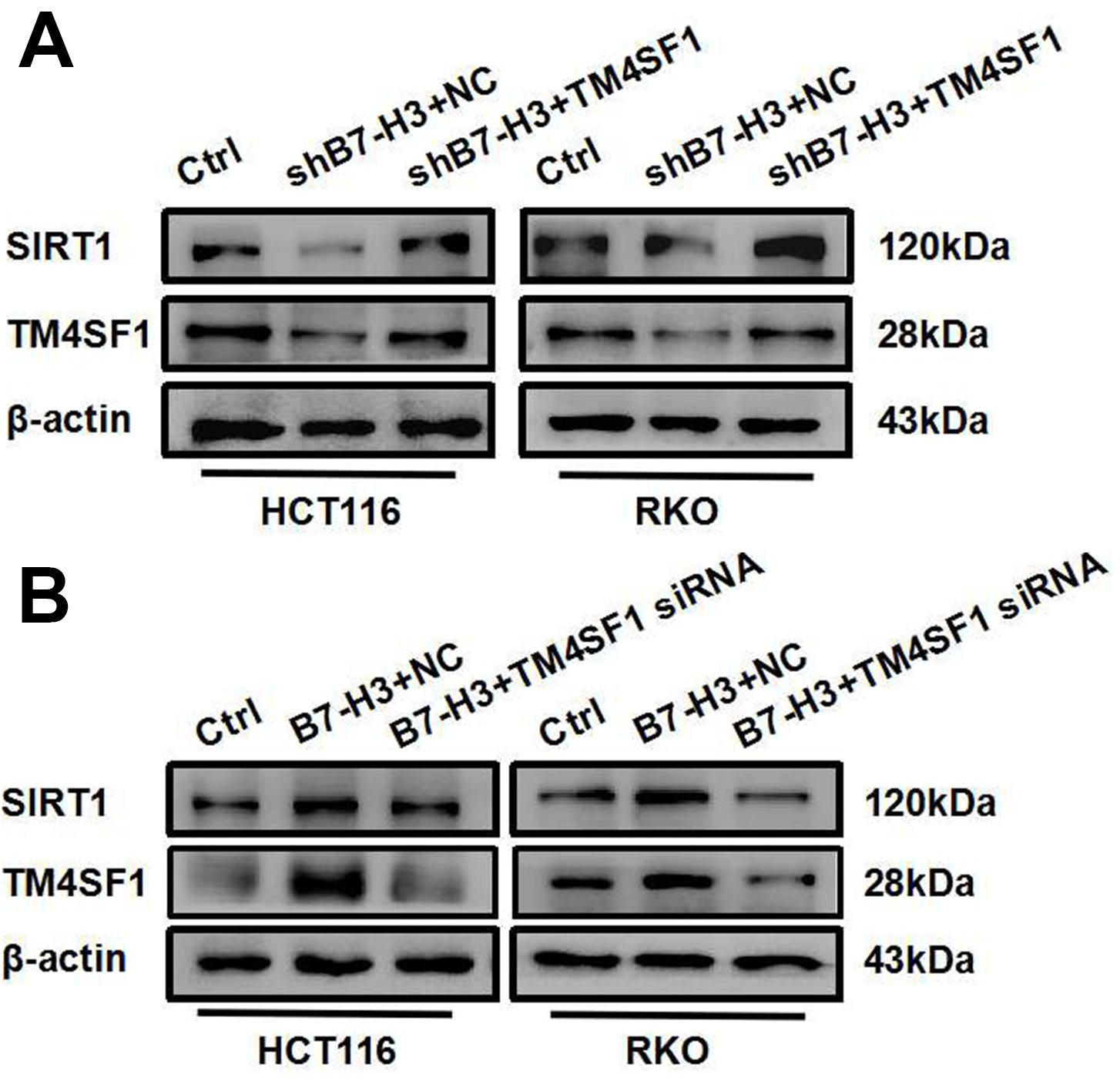

Supplement: Supplementary file 6 — Figure S5 [file 41419_2021_3736_MOESM6_ESM.jpg]

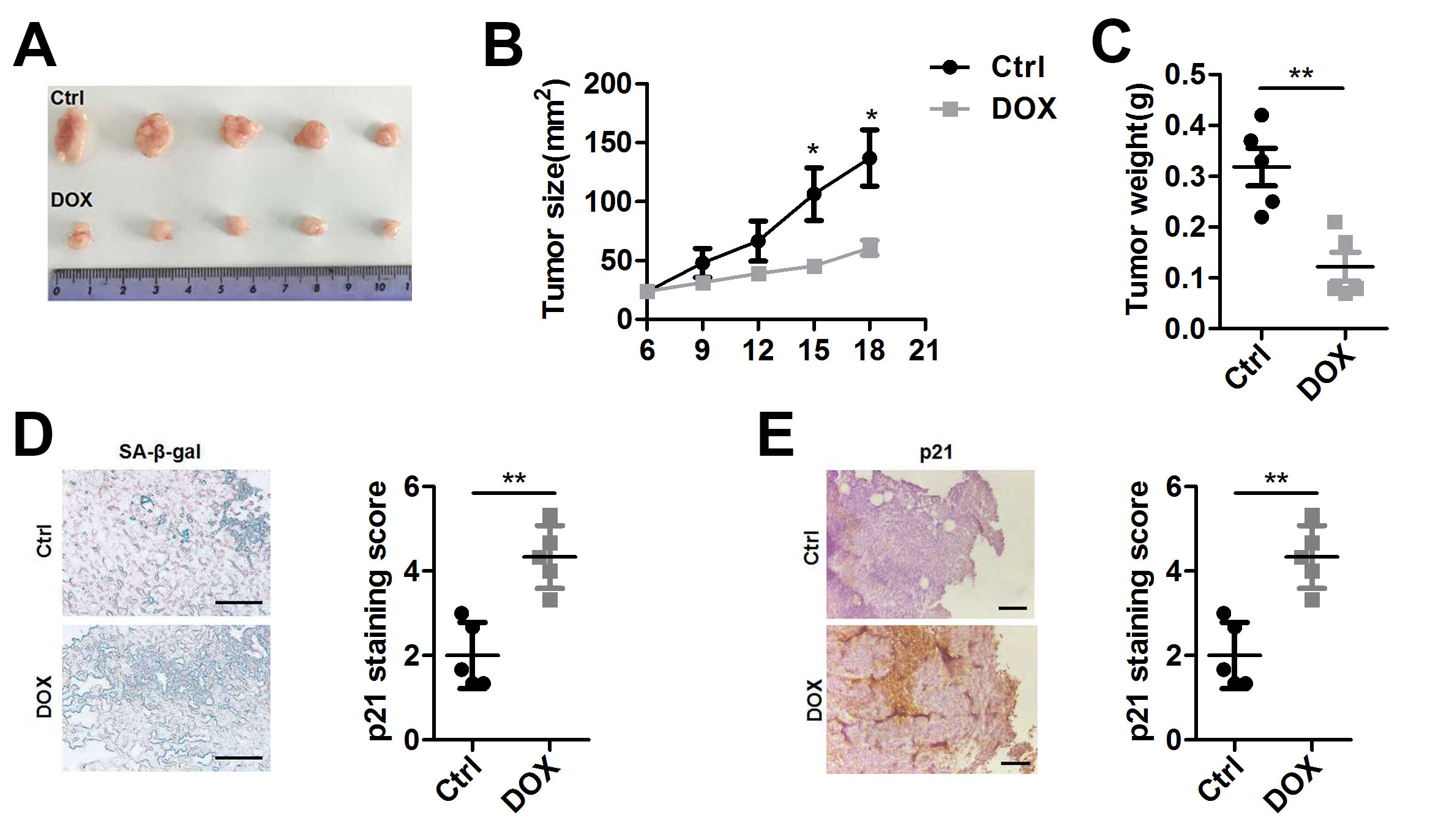

Supplement: Supplementary file 7 — Figure S6 [file 41419_2021_3736_MOESM7_ESM.jpg]

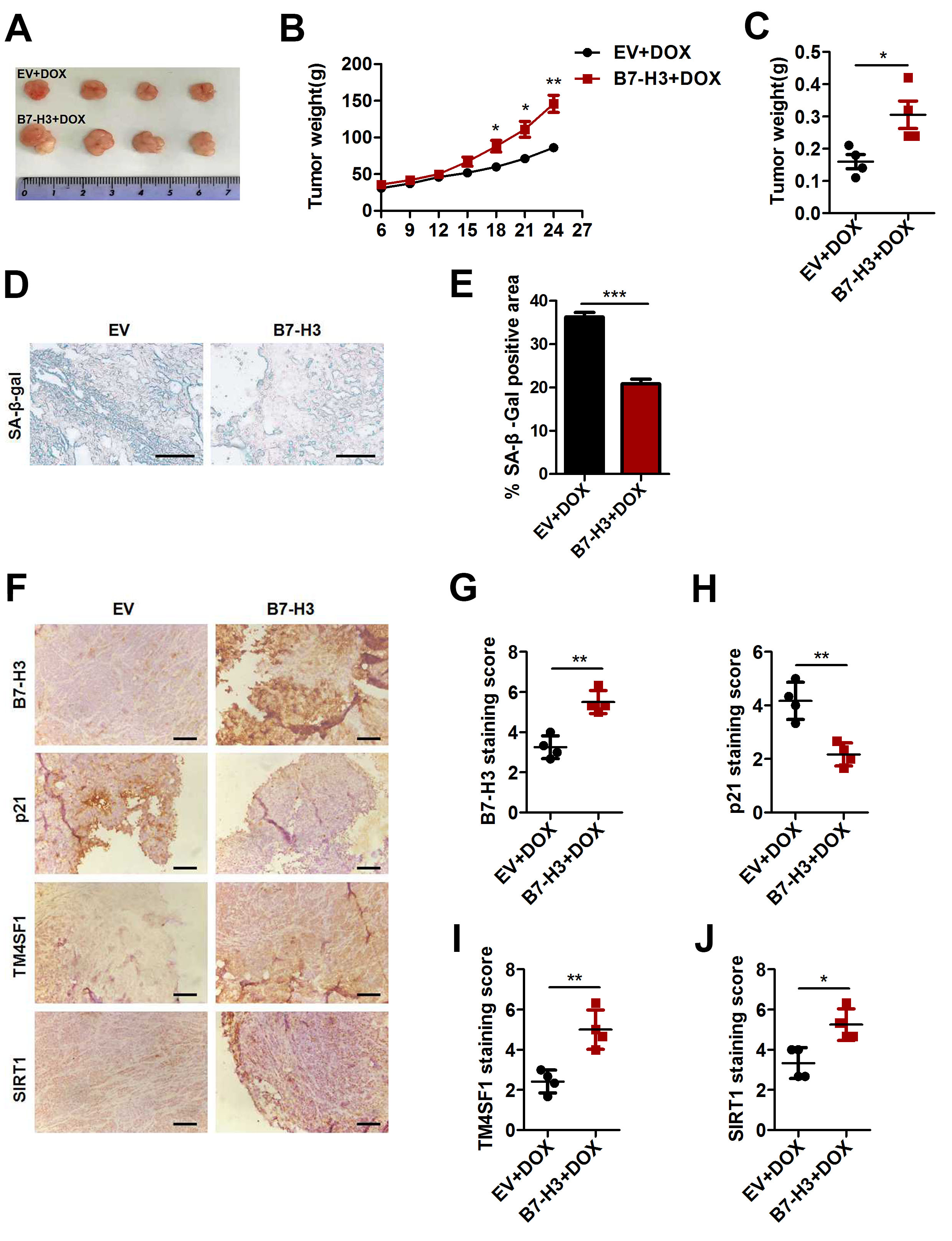

Supplement: Supplementary file 8 — Figure S7 [file 41419_2021_3736_MOESM8_ESM.jpg]
